# Spring loading a pre-cleavage intermediate for hairpin telomere formation

**DOI:** 10.1093/nar/gkv497

**Published:** 2015-05-24

**Authors:** Danica Lucyshyn, Shu Hui Huang, Kerri Kobryn

**Affiliations:** Department of Microbiology & Immunology, College of Medicine, University of Saskatchewan, Academic Health Sciences Building, 107 Wiggins Road, Saskatoon, SK S7N 5E5, Canada

## Abstract

The *Borrelia* telomere resolvase, ResT, forms the unusual hairpin telomeres of the linear *Borrelia* replicons in a process referred to as telomere resolution. Telomere resolution is a DNA cleavage and rejoining reaction that proceeds from a replicated telomere intermediate in a reaction with mechanistic similarities to that catalyzed by type IB topoisomerases. Previous reports have implicated the hairpin-binding module, at the end of the N-terminal domain of ResT, in distorting the DNA between the scissile phosphates so as to promote DNA cleavage and hairpin formation by the catalytic domain. We report that unwinding the DNA between the scissile phosphates, prior to DNA cleavage, is a key cold-sensitive step in telomere resolution. Through the analysis of ResT mutants, rescued by substrate modifications that mimic DNA unwinding between the cleavage sites, we show that formation and/or stabilization of an underwound pre-cleavage intermediate depends upon cooperation of the hairpin-binding module and catalytic domain. The phenotype of the mutants argues that the pre-cleavage intermediate promotes strand ejection to favor the forward reaction and that subsequent hairpin capture is a reversible reaction step. These reaction features are proposed to promote hairpin formation over strand resealing while allowing reversal back to substrate of aborted reactions.

## INTRODUCTION

An unusual but elegant solution to the end-replication-problem for linear replicons is represented by hairpin (hp) telomeres, covalently closed DNA hairpins at the ends of the linear DNA. Amongst bacteria, linear replicons with hp telomeres are found in the species of the genus *Borrelia*, in *Agrobacterium tumefaciens* and in a handful of bacteriophages that maintain a linear plasmid prophage (1–4). The hp telomeres present to the replication machinery an uninterrupted DNA chain. Bidirectional replication from an internal origin produces, after replication through the hairpin telomeres, replication intermediates with replicated telomere (*rTel*) junctions possessing inverted repeat sequence symmetry (5–8). The resulting daughter DNA molecules are covalently linked via the *rTel* junctions and must be separated by a specialized DNA breakage and reunion reaction, referred to as telomere resolution, that reforms the hp telomeres to allow for subsequent segregation (7,9–10). The essential, specialized telomere resolvase that performs this reaction for *Borrelia* is known as ResT (11,12). The characterized telomere resolvases of *A. tumefaciens* and the phage systems are often named ‘protelomerases’.

The telomere resolvases share a common mode of DNA cleavage and rejoining with similarities to the mechanism of phosphoryl transfer found in the Type IB topoisomerase and tyrosine recombinase families of enzymes (8). DNA cleavages occur 6 bp apart at the symmetry axis of the *rTel* and the resulting transient cleavage intermediate possesses the telomere resolvase covalently linked to the cleaved strands via 3′-phosphotyrosyl bonds and 6 nt self-complementary 5′-overhangs that must be refolded to effect strand transfer to reseal the DNA into hp telomere products (3,7,13–14).

**Figure 1.**
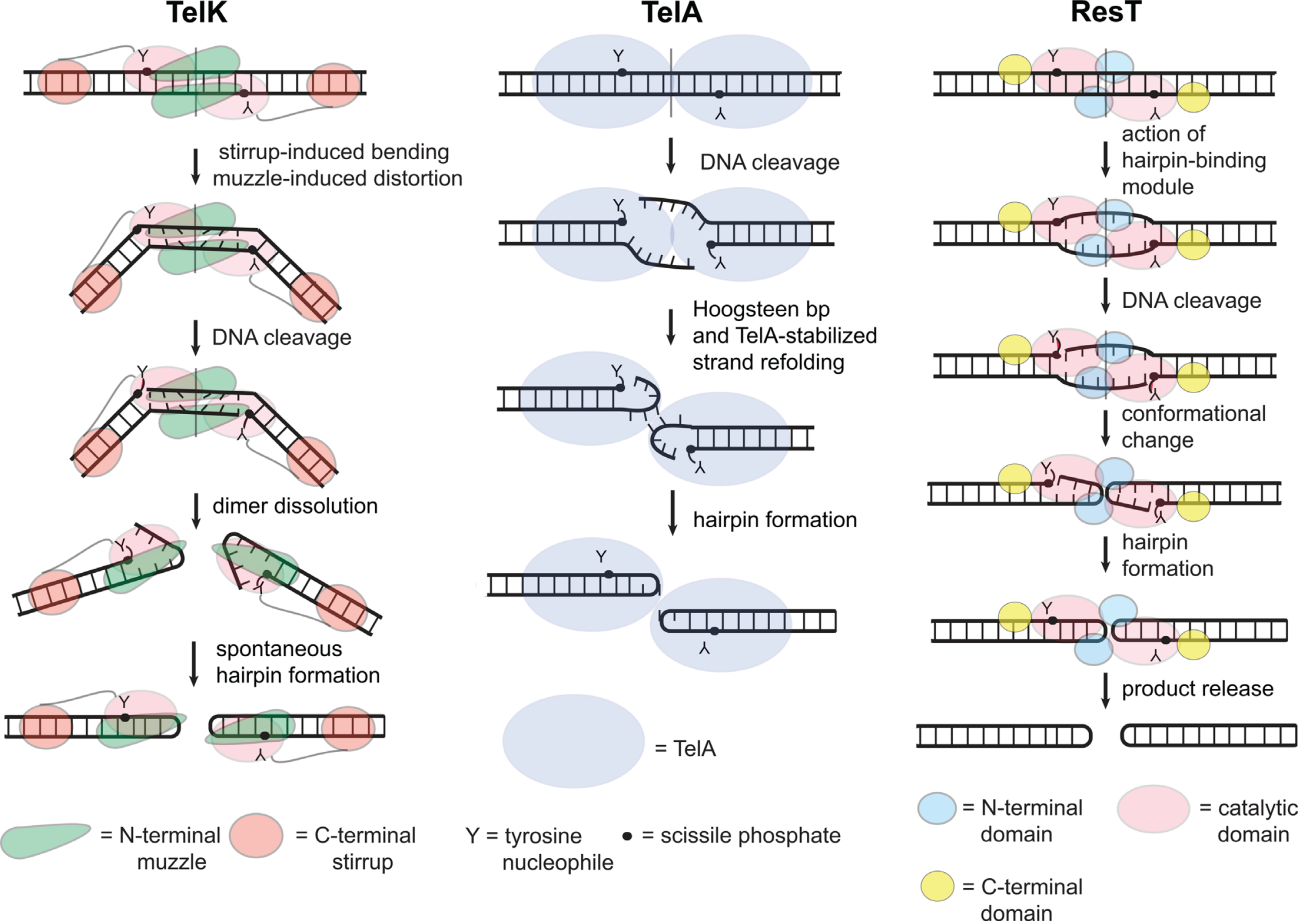
Models of telomere resolution derived from different systems. Schemata of the key steps in the reaction for TelK, TelA and ResT are shown. **TelK:** binding to the substrate DNA induces a large bend in the DNA that features a displacement of the helical path at the dimer interface, severely buckling the basepairs between the scissile phosphates. This proposed pre-cleavage intermediate ‘spring loads’, or stores the energy needed to drive, upon subsequent DNA cleavage, dimer dissolution and spontaneous hairpin formation. The energy stored in the pre-cleavage intermediate, and stable binding of TelK to the final hairpin telomere products, are factors hypothesized to drive the reaction forward. Support for this model is derived from the phenotype of a deletion mutant of the stirrup domain and from the observed non-turnover of the enzyme. Deletion of the stirrup domain, which stabilizes the DNA bend by interacting with the substrate DNA at the outer extremes of the *rTel*, left TelK competent to reversibly cleave the substrate but not to form the hairpins (3,17). **TelA:** post-cleavage stabilization of strand refolding, via TelA–DNA contacts and Hoogsteen basepairing across the dimer axis, and stable binding of TelA to the final hairpin telomere products are factors hypothesized to drive the reaction forward. The whole reaction occurs in the context of a dimer of TelA. Support for this model comes from the dimeric TelA-hp product complex, the behavior of TelA mutants in sidechains implicated in stabilizing the refolding strands and from the sensitivity of telomere resolution to *rTel* sequence changes that would interfere with the stabilizing Hoogsteen base pair formation (16). **ResT:** initial substrate binding and dimerization engages the hairpin-binding module at the end of the N-terminal domain of ResT to distort the DNA between the scissile phosphates allowing DNA cleavage by the catalytic domain. This distortion has been variously visualized as pre-formation of the hairpins (23) or more simply as DNA unwinding (15). An uncharacterized strand refolding ensues that captures the hairpin conformation after strand sealing; this occurs in the context of an intact dimer of ResT (15). Hairpin telomere products are released after both hp telomeres are formed (15). Support for this proposed model is derived from the behavior of hairpin-binding module mutants and from studies of product release in reactions with bead-immobilized *rTel*s (15,23).

Less well understood are the mechanism of strand refolding that produces the hairpins and the determinants of reaction directionality. The available data support conflicting models in different systems. The competing models differ most markedly in whether they hypothesize enzyme-mediated strand refolding in the context of the dimer, or a spontaneous hairpin formation following DNA cleavage and enzyme dimer dissolution. Models of telomere resolution by *Borrelia burgdorferi* ResT (15) and the *A. tumefaciens* telomere resolvase, TelA (16), involve enzyme-mediated strand refolding, while that of the *Klebsiella oxytoca* phage ϕKO2 telomere resolvase, TelK (17), features spontaneous strand refolding (Figure 1).

DNA processing enzymes that use a topoisomerase-like mechanism, that features transient covalent protein-DNA cleavage intermediates, keep the reacting DNA partners together until reaction completion. This avoids the formation of abortive reaction products. In the case of telomere resolution, abortive reaction products would be highly deleterious DNA double-strand breaks at the end of the linear chromosome or plasmids (15). Each DNA cleavage and rejoining step in telomere resolution is, in principle, reversible. Furthermore, while the chemistry of DNA cleavage and joining is isoenergetic, the hairpin products of the reaction are a higher energy form than the starting substrate. This is due to the fact that it is not possible for nucleic acid hairpins to have complete base pairing (17–19). These features make it difficult to identify the determinants of reaction directionality. It is clear that telomere resolution requires the base pairing between the scissile phosphates of the substrate *rTel* to be disrupted, for the cleaved strands to be refolded into hairpins and for this process to proceed through stabilized reaction intermediate(s) that promote the formation of products in preference to regeneration of substrate. Different models have been proposed to meet these requirements (Figure 1).

In the present study, we investigated the twin issues of reaction directionality and hp telomere formation for telomere resolution catalyzed by ResT. By use of substrate modifications that promote destabilization of duplex DNA we present evidence that ResT forms and stabilizes an underwound conformation of the *rTel* that promotes DNA cleavage and drives the reaction forward to hp formation. Furthermore, we characterize ResT catalytic domain mutants that are defective for telomere resolution on unmodified substrates that are rescued by substrate modifications that mimic an underwound conformation of the *rTel*. Contrary to our former conception of the reaction, in which the hairpin-binding module at the end of the N-terminal domain promotes substrate distortions that allow DNA cleavage by catalytic domain, rendering the catalytic domain a passive module, a picture emerges of a collaboration of hairpin-binding module and catalytic domain sidechains that generates and stabilizes an underwound ‘spring loaded’ pre-cleavage intermediate. This intermediate is proposed to be primed to promote ejection of the cleaved strands promoting the forward trajectory of the reaction, favoring hairpin telomere formation in preference to regeneration of substrate. Intriguingly, some of these same residues are also implicated in surveillance of base pair formation during strand joining to generate the hairpin telomeres. This renders hp telomere capture a reversal process if strands lacking dyad symmetry are being refolded.

## MATERIALS AND METHODS

### Proteins and oligonucleotide substrates

All oligonucleotides were purchased from Integrated DNA Technologies, except for the OPS modified oligonucleotide, which was synthesized by A.B. Burgin (deCode BioStructures Bainbridge Island, WA, USA) as described in ((20,21); Supplementary Table S1). The missing base oligonucleotides use IDT's 1′,2′-dideoxyribose (dSpacer) modification and were obtained as polyacrylamide gel electrophoresis (PAGE)-purified oligonucleotides (Supplementary Material and Methods). All reactions used ResT variants with an N-terminal (6X -His) protein tag that were purified as reported in (22).

### Telomere resolution assays

**Figure 2.**
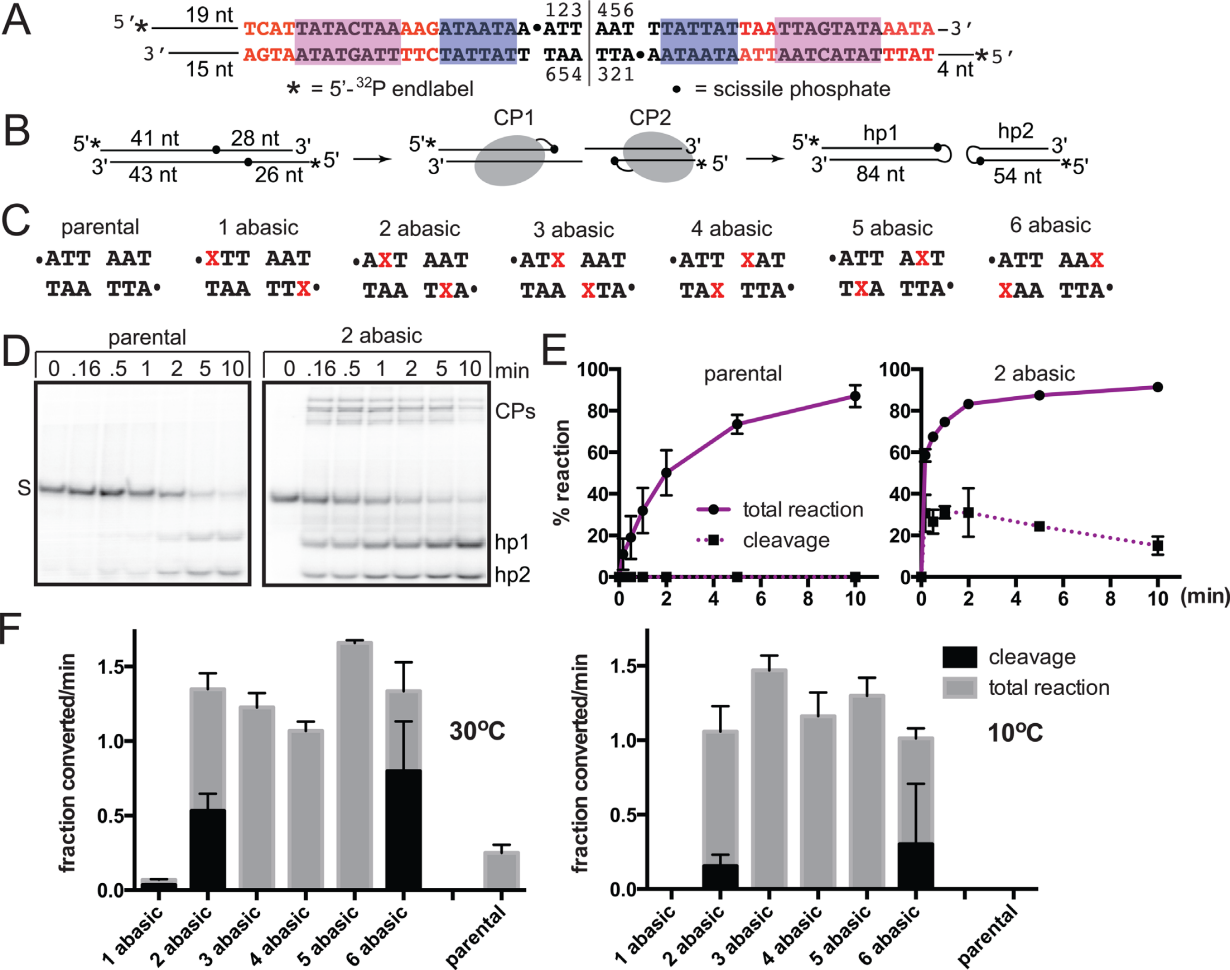
Missing base modifications between the scissile phosphates of the substrate *rTel* alleviate the cold-sensitivity of telomere resolution. (**A**) The model Type 2 *rTel* used in this study. To aid the annealing of the oligonucleotides into an *rTel*, the normal dyad symmetry of native *rTel*s was broken by having distinct sequences on the left and right sides in positions where the sequence of different Type 2 *rTel*s vary (red letters). The right hand halfsite sequence is derived from the left telomeres of lp28-2 and lp36. The left hand halfsite sequence is derived from the right telomere of lp28-4 (25). The magenta and blue/shaded boxes highlight regions of conserved sequence. To allow differentiation of products from the left versus right side of the *rTel* 15 bp of nontelomeric sequence was added to the left side. The substrate nucleotide numbering noted for the unmodified parental substrate follows the convention reported in the TelA–DNA and TelK–DNA structures. The vertical line between nucleotides 3 and 4 denotes the dyad symmetry axis of the *rTel*. (**B**) Schematic representation of a telomere resolution reaction with 5′-^32^P endlabeled substrate. DNA chain lengths, from the substrate ends to the scissile phosphates, are indicated in the substrate; chain lengths in the hairpin telomere products are shown. Cleavage products (CP), in which ResT is covalently linked to the cleaved DNA via a phosphotyrosyl bond, can be visualized in reactions where hairpin telomere formation fails or is inefficient. (**C**) Sequence between the scissile phosphates of the parental substrate and the range of tested *rTels* with missing base (abasic) sites in symmetrically located positions between the scissile phosphates. The abasic modifications are represented in the sequence by the red/shaded X's and the scissile phosphates by dots. (**D**) Representative 8% PAGE 1× TAE/0.1% SDS gels of timecourse reactions with ResT, parental *rTel*and the 2 abasic *rTel* detailed in (C). S, represents the mobility in the gel of the substrate; CPs, mark the gel migration position of the cleavage products (when detectable); hp1 & hp2, mark the gel position of the hairpin telomere products detailed in (B). (**E**) The left graph shows % reaction (DNA cleavage and total reaction) versus reaction time is plotted for a telomere resolution reaction with wild type ResT and parental *rTel* incubated at 30°C (left graph). In the reaction shown the cleavage intermediates do not accumulate and are not detected on the gel so total reaction is equivalent to the rate of hairpin telomere formation. When cleavage intermediates do accumulate the rate of their formation was determined separately and the total reaction becomes the summed rate of DNA cleavage plus hp telomere formation. The right graph shows % total reaction versus reaction time of a telomere resolution reaction with wild type ResT and the 2 abasic *rTel* incubated at 30°C. Shown are the mean and standard deviation of at least 3 independent replicates. Initial rates shown in (F) were determined from individual % reaction versus time plots by determination of the slope of the initial linear portion of the curves; means and standard deviations for the initial rates were determined from at least three independent timecourses. (**F**) Comparison of the initial rates of DNA cleavage and total reaction (DNA cleavage + hp telomere formation) of the parental *rTel* and the abasic modifications shown in A and C) in reactions incubated at the standard reaction temperature of 30°C compared to incubations at 10°C. The initial rates are expressed as the fraction substrate converted/min (1.0 being 100% conversion). Shown is the mean and standard deviation of at least three independent replicates. The asterisk above the one abasic data indicates that the slow observed DNA cleavage was aberrant producing, predominantly, substrate cleaved on only one strand.

Timecourse telomere resolution assays were performed in 120 μl master mixes at the temperature indicated in the experiment with 630 fmol of 5′-^32^P radiolabeled substrates (5.25 nM) and 75 nM ResT in reaction buffer (25 mM Tris–Cl, pH 8.5; 1 mM ethylenediaminetetraacetic acid (EDTA), pH 8.0; 100 μg/ml bovine serum albumin; 100 mM NaCl). Eighteen microloters aliquots were removed into sodium dodecyl sulphate (SDS) load dye to a 1× final concentration. 1× SDS load dye contains 20 mM EDTA, 3.2% glycerol, 0.1% SDS and 0.0024% bromophenol blue. 10 s, 30 s, 1 min, 2 min, 5 min and 10 min time points or 30 s, 2 min, 5 min, 10 min, 20 min and 30 min time points were taken for initial rate determinations, depending upon the results of preliminary timecourse reactions. Electrophoretic analysis was performed on 20 cm × 20 cm 8% PAGE 1× Tris-acetate EDTA (TAE)/0.1% SDS gels electrophoresed at 13 V/cm for 2 h. The gels were dried and exposed to phosphor screens and analysis on a BioRad FX phosphorimaging machine. Reactions were performed, at a minimum, in triplicate and the data were quantified with BioRad's Image Lab software. Gel exposures below the software's pixel saturation level were used and quantified with local background subtraction. The gels were quantified by summing the band intensities in each lane and calculating the proportion contributed by cleavage intermediates and hp telomere products, respectively. Graphs and reaction statistics were generated with Prism's GraphPad 6.0. Representative gels, reaction curves and details for initial rate determinations are found in Figure 2 and Supplementary Figure S4.

Denaturing gel analysis, where used, was performed with 18 μl aliquots removed into 18 μl of formamide load dye (2× = 95% formamide, 20 mM EDTA (8.0), 0.025% bromophenol blue) then heated at 95°C for 6 min prior to loading onto an 8% PAGE 1× Tris borate EDTA (TBE)/6 M urea sequencing gel pre-run for 1 h at 36 W. The gel was run at 30 W for 1 h and then the gel was transferred to Whatman 3MM paper and dried prior to exposure to a phosphor screen.

## RESULTS

### Mimicking substrate unwinding can alleviate the cold-sensitivity of telomere resolution

Previous results suggest that ResT needs to distort the replicated telomere (*rTel*) in some fashion between the scissile phosphates to allow DNA cleavage and subsequent hp formation (see Figure 1 and (23)). Those results, combined with the presence of unpaired nucleotides in the TelA–DNA product complex, and extrahelical bases stabilized by stacking contacts with TelA in the TelA–DNA ‘refolding intermediate’ (16), prompted us to examine the effect on telomere resolution of substrate DNA modifications that mimic unwinding of the sequence between the scissile phosphates. Our analysis started with an examination of the effect, on telomere resolution, of using substrates with symmetrically positioned missing base modifications. We chose for our parental sequence a Type 2 *rTel*, a type of telomere that is permissive of different DNA sequences between the scissile phosphates, to avoid confounding effects of a requirement for site-specific contacts between ResT and this part of the substrate DNA (14,24–25). Missing base modifications have the dual effects of eliminating partners for base pairing and stacking, thereby, destabilizing the double helix (26). They can also reduce the need for enzyme stabilization of extrahelical bases that may transiently arise in DNA transactions that involve duplex unwinding (27–29). DNA transactions that feature a DNA unwinding step often display a marked reduction in the efficiency of unwinding at reduced temperatures. This cold-sensitivity is due to the reduced ability of the reaction milieu to contribute energy to transiently melt the DNA. A classic example of such cold-sensitivity in a DNA unwinding transaction, promoted without the use of ATP hydrolysis, is ‘open complex’ formation at the −10 element of bacterial promoters. Substrate modifications that destabilize the duplex DNA of the −10 element can alleviate the cold-sensitivity of open complex formation (28).

If telomere resolution possesses an underwound, but not yet cleaved, intermediate then one would expect missing base modifications to reduce the cold-sensitivity of telomere resolution. This would be expected by virtue of the fact that the base pairs at the sites of the missing base modifications have been eliminated. In Figure 2 we present an analysis of the effect on the initial rate of telomere resolution of missing base modifications positioned between the scissile phosphates of the substrate. Reaction rates obtained at 30°C and 10°C are compared. An unmodified ‘parental’ *rTel* and an array of *rTels* with missing bases, that include removal of the bases at positions 1–6 in the substrate, were tested (see Figure 2A and C for the nucleotide numbering convention and substrate details). Removal of bases at the 1 position eliminates the base step around the scissile phosphates producing an *rTel* with poor reactivity; the slow reaction we did observe with this substrate consists chiefly in the aberrant production of *rTel* cleaved on only one strand (Figure 2F and data not shown). All the other missing base substrates do allow reaction and afford a 3–5-fold stimulation of the reaction rate at 30°C relative to the parental *rTel*. Lowering the reaction temperature to 10°C results in little effect on the reaction rate of the missing base substrates but abolishes the reactivity of the unmodified *rTel* (see Figure 2F).

The missing base modifications at position 2 and position 6 (2 abasic and 6 abasic in Figure 2C) result in the accumulation of cleavage intermediates in which the *rTel* has been cleaved but not yet hairpinned, an intermediate that is normally too transient to see with unmodified substrates. This effect is consistent with missing bases at these positions promoting DNA cleavage but somewhat compromising strand joining, either to produce hp telomere products or to regenerate the substrate *rTel*. The substrate with missing bases at position 5 (5 abasic) disrupts the same basepairs as the two abasic *rTel* but does not show similar accumulation of cleavage products (Figure 2F). This suggests that the presence of the nucleotides at position 2 is important for strand joining.

**Figure 3.**
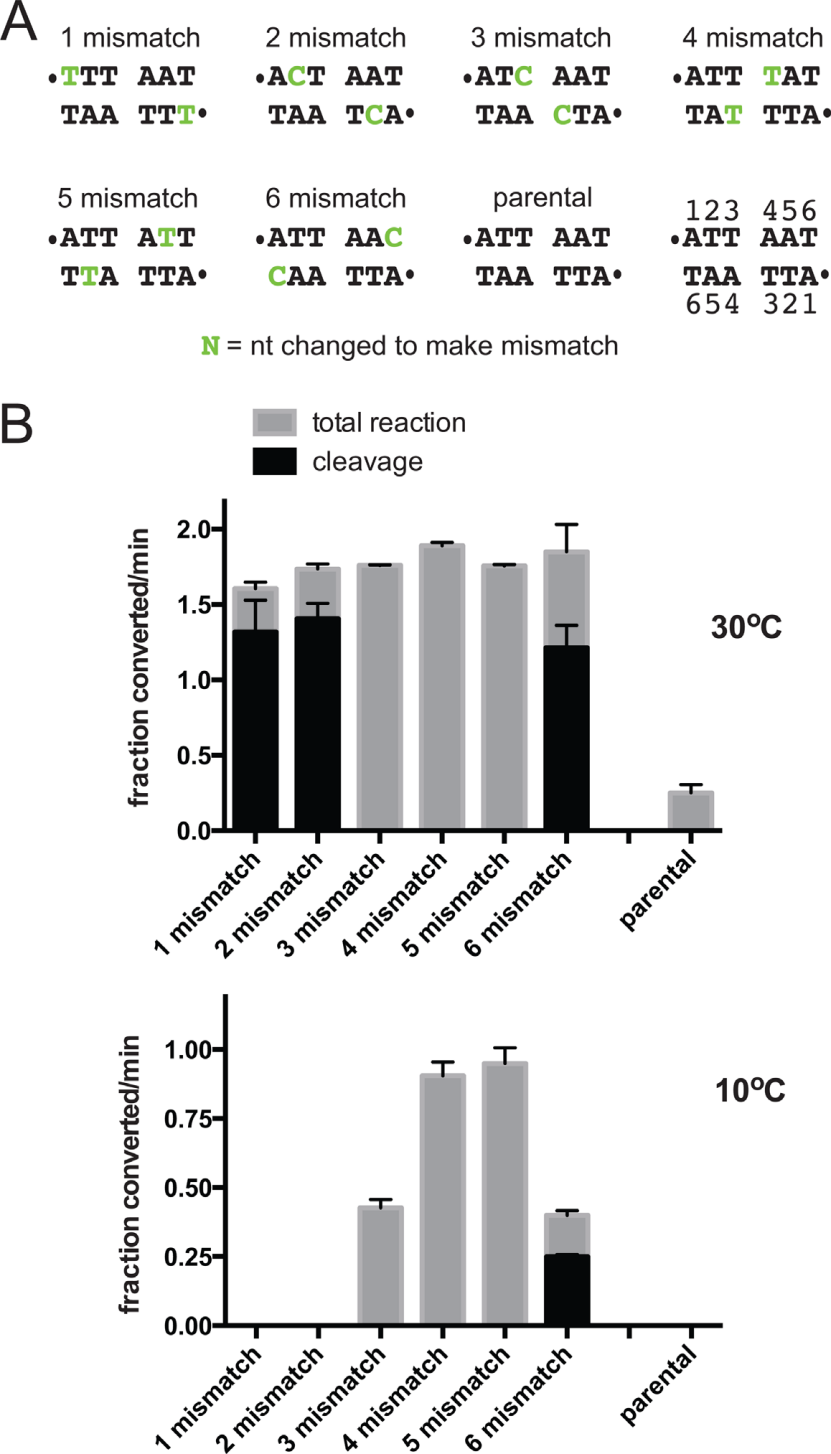
The effect of DNA mismatches, between the scissile phosphates of the substrate, on the cold-sensitivity of telomere resolution. (**A**) Sequence between the scissile phosphates of the parental substrate and the range of tested *rTels* with mismatches in symmetrically located positions between the scissile phosphates. The parental *rTel* and substrate nucleotide numbering is shown. The sequence changes that introduce the mismatches are represented in the sequence by the green/shaded letters and the scissile phosphates by dots. (**B**) Comparison of the initial rates of DNA cleavage and total reaction (DNA cleavage + hp telomere formation) of the parental *rTel* and the substrates with mismatches shown in (A) in reactions incubated at the standard reaction temperature of 30°C versus 10°C. The initial rates are expressed as the fraction substrate converted/min (1.0 being 100% conversion). Shown is the mean and standard deviation of at least three independent replicates.

Missing base modifications disrupt substrate base pairing but may also remove important base stacking interactions or contacts with ResT required for the reaction. Therefore, our analysis continued with an examination of the effect on the initial rate of telomere resolution of mismatches positioned between the scissile phosphates of the substrate, comparing the reaction rates obtained at 30°C and 10°C (Figure 3). Similar to the effect of missing base modifications, substrates with mismatches show stimulation of the reaction rate at 30°C relative to the parental *rTel*. The alleviation of the cold-sensitivity of telomere resolution was less pronounced and less general than that noted with the missing base modifications. Substrates with mismatches at positions 1 or 2 (1 mismatch and 2 mismatch; Figure 3A) were unreactive at 10°C. Substrates with mismatches at positions 4 or 5 (4 mismatch and 5 mismatch; Figure 3A) show the greatest relief of cold sensitivity (Figure 3B). Where operative, the reduction in cold-sensitivity was due to the mismatches, *per se*, and not due to the change of nucleotide. This was shown by testing mutant substrates with compensatory mutations that restore base pairing but maintain the sequence change present in the mismatch *rTel*s tested in Figure 3; resolution of these control mutant *rTel*s remains cold-sensitive (Supplementary Figure S1).

Substrates that change the bases at position 1, 2 or 6 to create the mismatches showed a marked accumulation of cleavage intermediates rather than direct conversion to hp telomeres. This is consistent with a stimulation of DNA cleavage accompanied by an impaired ability to rejoin the cleaved strands. However, the substrate with changed bases at position 5 (5 mismatch) that disrupts the same base pairs as in the 2 mismatch *rTel*, showed no accumulation of cleavage intermediate (Figure 3B). The effect of compromised strand rejoining was due to the mismatches. Substrates with compensatory mutations that restore base pairing did not accumulate cleavage intermediates (Supplementary Figure S1). The position specific effects on strand rejoining may be indicative of specific contacts between ResT and the *rTel* at positions 1 and 2. A precedent for this is revealed in the structure of the *A. tumefaciens* telomere resolvase, TelA, where TelA ‘reads out’ the hp telomere by making base-specific contacts with the T1 and C2 nucleotides (16). The disparate results between the 1 mismatch and 1 abasic *rTel*s, with the former supporting cleavage and the latter being almost unreactive, lends further support to our contention that ResT may need to make a contact with a base at position 1.

### Characterization of ResT mutants rescued by missing base modifications of the *rTel*

Previous studies of a region in ResT with similarity to the ‘YREK’ motif of IS4 transposases, that stabilize the hairpin intermediate of transposon excision, implicated this ‘hairpin-binding module’ in ResT in substrate DNA distortions needed for DNA cleavage and hp formation. A deficiency in telomere resolution for most mutants in the ResT hairpin-binding module is only apparent at reduced reaction temperature. This cold-sensitivity was rescued by heteroduplexing the central two nucleotides of the *rTel* (23). The strongest hairpin-binding module mutant characterized (P139AW141A) was found to be strongly defective at the normal reaction temperature. This mutant was also partially rescued by heteroduplex modification (23). In order to determine if mutants in other regions of ResT display a similar phenotype we regularly assayed new mutants for a similar phenotype. We performed a mutagenic survey of all the histidine and glutamine residues in ResT that are conserved across both relapsing fever and Lyme disease causing *Borrelia* species. Additionally, we made mutants in ResT that are predicted to correspond to loops, in the structures of the TelA–DNA and TelK–DNA complexes, that track along opposite grooves near the scissile phosphates. We suspected these loops may be appropriately positioned to stabilize the hypothesized underwound pre-cleavage intermediate. For inclusion in this study, we screened for mutants that were inactive at the standard reaction temperature of 30°C that were rescued by substrate modifications that destabilize the duplex DNA between the scissile phosphates (see Supplementary Material and Methods and Supplementary Figure S2 for details of the mutants examined and how they were screened). By analogy to the rescue of ‘DNA unwinding region’ mutants of sigma factor, by destabilized promoter substrates (28,30), we reasoned that ResT mutants that met our screening criteria would likely be involved in forming the hypothesized underwound pre-cleavage intermediate.

Examination of DNA binding activity of the mutants that met these criteria indicated that their global structure was not compromised (Supplementary Figures S2 and S3). The majority of the mutants were found to be in the catalytic domain (V231A, E325A, D328A and H334A). Only one hairpin-binding module mutant, in addition to the previously characterized P139AW141A mutant, was identified as inactive at normal reaction temperature but rescuable by substrate modifications (Q161A). This indicates that the hairpin-binding module and the catalytic domain cooperate to form the underwound pre-cleavage intermediate rather than the catalytic domain playing a passive role restricted to catalyzing the chemistry of DNA cleavage and rejoining.

**Figure 4.**
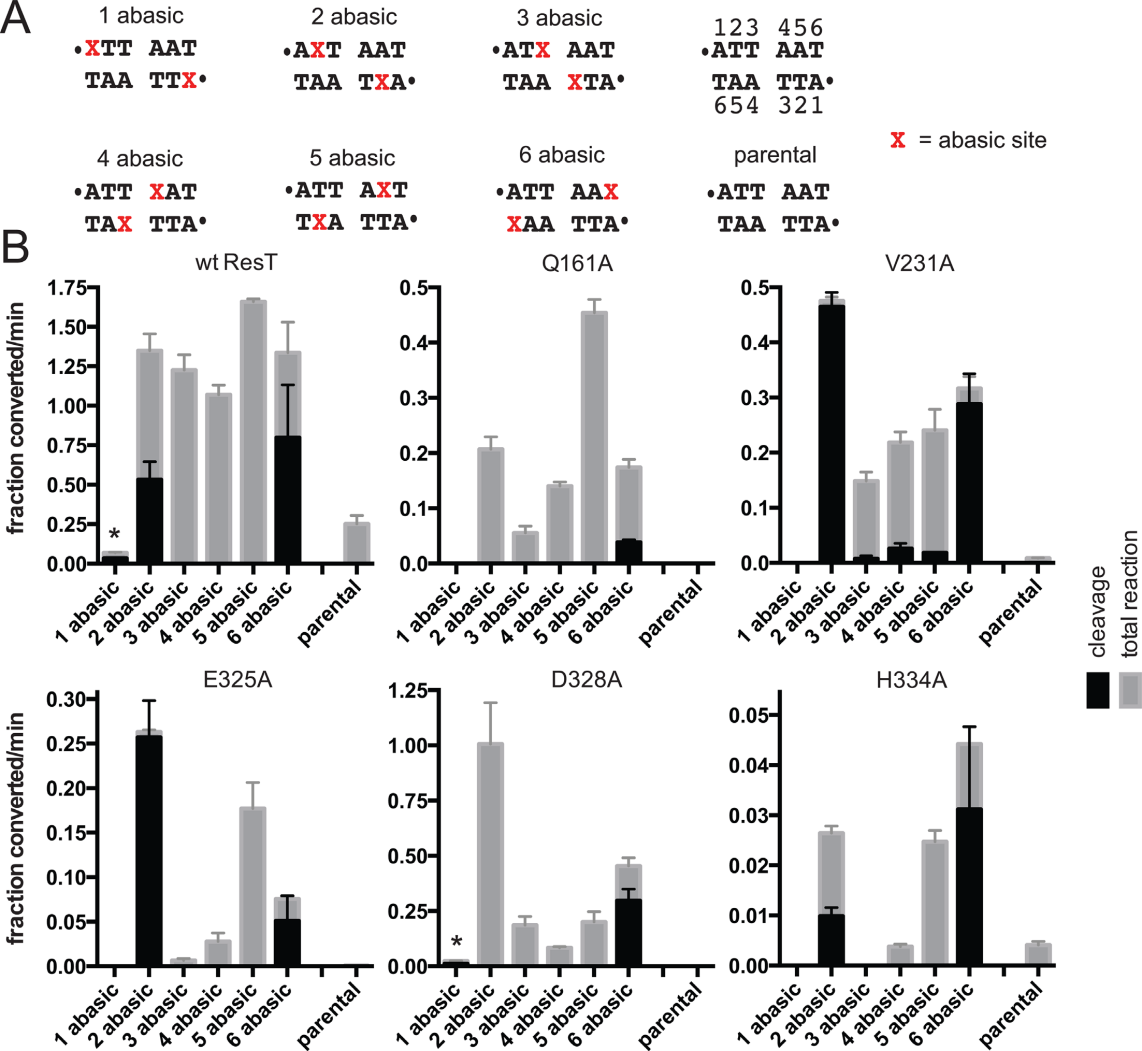
The pattern of activity of ResT mutants defective for telomere resolution assayed with substrates with missing base modifications. (**A**) Sequence between the scissile phosphates of the parental substrate and the range of tested substrate *rTels* with missing base (abasic) sites positioned in symmetric locations on the top and bottom strands (details as in Figure 2C). (**B**) Comparison of the initial rates of DNA cleavage and total reaction (DNA cleavage + hp telomere formation) of wild-type ResT and the indicated mutants assayed on the parental Type 2 *rTel* and the array of abasic *rTel*s detailed in (A). The x-axis presents the substrates assayed and the y-axis the initial rates of DNA cleavage (black bars) and the total reaction rate (DNA cleavage + hp formation, gray bars). The initial rates are expressed as the fraction substrate converted/min (1.0 being 100% conversion). Normally, cleavage and hp formation are concerted, so no cleavage intermediates accumulate; in these cases the total reaction rate is just the rate of hp formation. In some cases, the missing base modifications allowed DNA cleavage with only slow, subsequent, hp telomere formation; this leads to the accumulation of cleavage intermediates (see Figure 2B schematic). Representative data and details of the initial rate determinations are shown in Supplementary Figure S4. The asterisk above the 1 abasic data (wt ResT and D328A reactions) indicates that the observed slow DNA cleavage was aberrant producing, predominantly, substrate cleaved on only one strand. All initial rates were determined from a minimum of three independent replicates, and the error bars represent the standard deviation. Note that the scaling of the y-axes are not necessarily the same from one ResT variant to another.

Telomere resolution assays with wild type ResT and the mutants, using the unmodified *rTel* versus missing base variants, are documented in Figure 4B. Details of the initial rate determinations are presented in Supplementary Figure S4. Alanine mutants at positions Q161, V231, E325 and D328 showed good rescue with some of the missing base modifications. Missing bases at position 2 gave optimal rescue of the V231A, E325A and D328A mutants; missing bases at position 5 gave optimal rescue of the Q161A mutant. Missing base modifications can reduce the need for enzyme stabilization of extrahelical bases that may transiently arise in DNA transactions that involve duplex unwinding (27–29). Mutants of residues in Tn5 transposase's ‘YREK’ motif, implicated in stabilizing a flipped out base that helps to form the DNA hairpin intermediate in that system, are rescued by a missing base modification at the position of the flipped out base (29,31). It is tempting to speculate that *rTel* bases at positions 2 and 5 may be, similarly, in an extrahelical/unwound conformation in the pre-cleavage intermediate, requiring stabilization via interaction with Q161, V231, E325 and D328. Further biophysical characterization will be needed to test this hypothesis.

The rescue afforded by the missing base modification at position 2 allows, predominantly, DNA cleavage but not hairpin formation for V231A and E325A, a hairpin formation defect greater than that seen with wild type ResT with this *rTel*. Unexpectedly, the reactions with Q161A and especially D328A show less accumulation of cleavage intermediate than reactions with wild type ResT (Figure 4B). This hints at a possibly enhanced ability of these mutants to form hp telomere without base pair formation near the site of strand joining.

### Assessing the cleavage competence of the mutants

**Figure 5.**
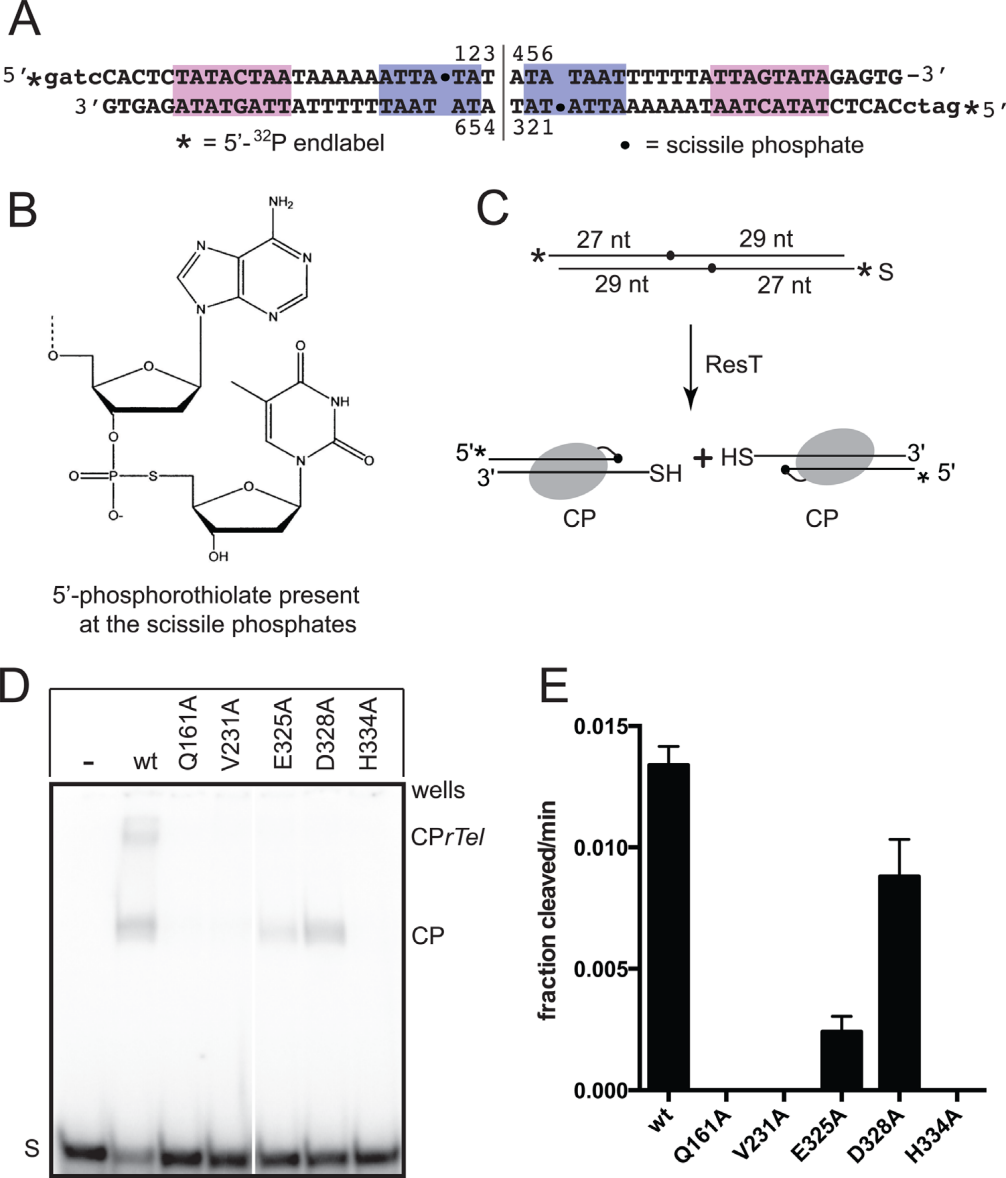
Assessing the cleavage competence of the mutants. (**A**) The OPS *rTel* used. The sequence of the *rTel* is derived from the left telomere of lp17. The magenta and blue/shaded boxes highlight regions of conserved sequence. The OPS *rTel* is dyad symmetric. This necessitates construction by annealing halfsites followed by ligation and gel purification (see Supplemental Material and Methods, Supplementary Table S1 and (23)). The substrate nucleotide numbering is noted. The vertical line between nucleotides 3 and 4 denotes the dyad symmetry axis of the *rTel*. The 5′-^32^P endlabels are indicated by asterisks, and the scissile phosphate are indicated by dots. (**B**) Schematic representation of the basestep surrounding the scissile phosphates in a suicide *rTel* harboring 5′-phosphorothiolate moieties (OPS). (**C**) Schematic of a replicated telomere cleavage assay performed with 5′-^32^P endlabeled OPS *rTel*. DNA chain lengths, from the substrate ends to the scissile phosphates, are indicated in the substrate. Cleavage products, in which ResT is covalently linked to the cleaved DNA, accumulate due to the inability of the resulting 5′-sulfhydryl groups to participate in strand joining reactions to generate hp telomeres or to regenerate substrate. S, represents the OPS *rTel*; CP, represents the cleavage products. (**D**) 8% PAGE 1X TAE/0.1% SDS gel analysis of reactions assayed with wild-type ResT and the telomere resolution deficient ResT mutants using an OPS-modified suicide *rTel*. Reactions containing 75 nM ResT, and 5.25 nM 5′-^32^P endlabeled OPS *rTel* were incubated at 30°C for 30 min. The ResT variant added is noted in the gel-loading key above the gel. S, represents the mobility in the gel of the OPS *rTel*; CP, represents the gel migration position of the cleavage products detailed in A); CP*rTel*, marks the gel migration position of a cleavage product in which only one strand has been cleaved. Note that the efficiency of OPS *rTel* utilization by ResT is less efficient than that of the unmodified ‘parental’ *rTel* used elsewhere in this study. (**E**) Comparison of the cleavage rate of the mutants compared to the cleavage rate of wild-type ResT. The initial rates are expressed as the fraction substrate cleaved/min (1.0 being 100% conversion). All rates are determined from a minimum of three independent replicates; the error bars represent the standard deviation.

Telomere resolution proceeds via a topoisomerase IB-like reaction mechanism that, in principle, features a reversible DNA cleavage step. It was, therefore, unclear whether the lack of reactivity of the mutants on unmodified *rTel* was due to a failure to initiate DNA cleavage or due to rapid reversal of the reaction back to substrate upon failure to complete the reaction. This issue was addressed by assaying the mutants with an *rTel* with 5′-phosphorothiolate (OPS) modifications at the scissile phosphates that allow DNA cleavage but do not support strand rejoining reactions, converting the OPS *rTel* into a suicide substrate (Figure 5B & C; (13,21)). The Q161A, V231A and H334A mutants were found to be unreactive with the OPS *rTel*. Similarly, the cleavage defective phenotype of hairpin-binding module mutants with an OPS *rTel* suggested that the hairpin-binding module mutants’ compromised activity on normal substrates was caused by an inability to start the reaction (23). By contrast, E325A and especially D328A were partially cleavage competent with the OPS *rTel* indicating that these mutants undergo abortive cycles of cleavage and strand rejoining on unmodified substrate (Figure 5D and E).

### The contribution of DNA bending to telomere resolution

**Figure 6.**
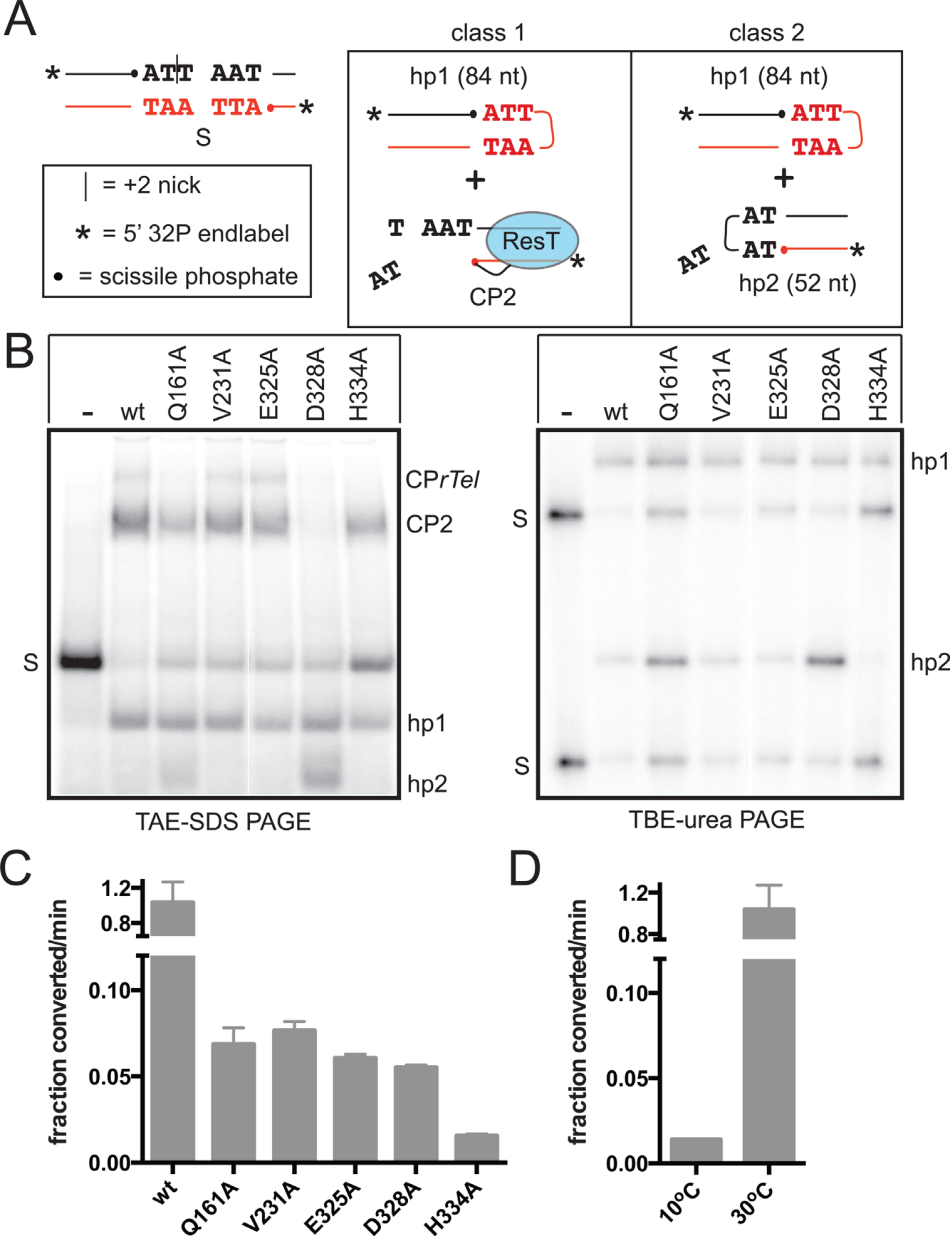
The pattern of activity of ResT mutants, defective for telomere resolution, assayed with a substrate nicked near the substrate's symmetry axis. (**A**) Schematic of the +2 nick *rTel*, sequence as depicted in Figure 2A. The two classes of reaction outcome observed are summarized schematically. In class 1, most of the substrate is cleaved normally, hp1 forms but hp2 formation is blocked or slow, resulting in the accumulation of cleavage product (CP2) in which ResT is covalently attached to the cleaved half site. In class 2, the nicked *rTel* is cleaved normally and both hairpin telomeres (hp1 and hp2) form. hp2, when it can form, does so despite being 2 nt shorter than normal, due to the diffusion away, after cleavage, of the dinucleotide 3′ to the top strand scissile phosphate and despite the fact that the resulting 4 nt strand lacks self-complementarity. (**B**) Summary 8% TAE–SDS and TBE–urea PAGE gels are shown of reactions of wild type and mutant ResT variants with the +2 nick Type 2 *rTel*. The reactions shown were incubated at 30°C for 30 min. Only free DNA strands enter the TBE–urea PAGE gels; the cleavage products with ResT covalently linked to the 5′-^32^P endlabeled strands do not enter these gels. S, represents the mobility in the gel of the substrate; CP2, marks the gel migration position of the cleavage product from the right side of the substrate; CP*rTel*, marks the gel migration position of a cleavage product in which only the top strand has been cleaved; hp1 and hp2, mark the gel position of the hairpin telomere products. (**C**) Comparison of the initial rates of total substrate usage of wild type and mutant ResT variants tested in reactions with nicked Type 2 *rTel* incubated at 30°C. All rates are determined from a minimum of three independent replicates; the error bars represent the standard deviation. (**D**) Comparison of the initial rate of total substrate usage of wild type ResT tested in reactions incubated at 10°C versus 30°C. No reaction was detectable for the mutants assayed at 10°C.

The effects of missing base modifications are potentially multifaceted. Missing base modifications provide a mimic of DNA unwinding mediated by bases assuming an extrahelical conformation. They also have the potential to promote DNA twisting/bending flexibility. Missing base modifications can also remove important specific contacts between enzyme and substrate (32,33). The proposed pivotal role of DNA bending in TelK-mediated telomere resolution (17) prompted us to examine the role of DNA bending. Nicks in the substrate can facilitate DNA bending without removing bases and only promote baseflipping or DNA melting immediately next to the nick (27,34–35). Therefore, to assess the relative contributions of DNA bending versus DNA unwinding, we sought to test the mutants with a substrate with a nick positioned between the scissile phosphates of the *rTel*. Preliminary tests were carried out with wild type ResT and substrates with nicks positioned at various positions between the scissile phosphates. Optimally reactive *rTels* were obtained by positioning the nick at the +2 or +4 positions (Supplementary Figure S5). The mutants were examined with the +2 nick substrate (Figure 6). Relative to the missing base modifications, only modest rescue of the reaction rate with the +2 nick modification of a Type 2 *rTel* is observed with the Q161A, V231A, E325A and D328A mutants; very little rescue of H334A mutant was observed (Figure 6B and C). These results indicate that increased flexibility of the substrate contributes to reaction rescue of the mutants but that this contribution is less important than the destabilization of the DNA double helix afforded by the missing base modifications. This conclusion is bolstered by the observation that the nick resulted in very little relief of the cold-sensitivity of telomere resolution with the wild type enzyme relative to that afforded by missing base modifications or mismatches between the scissile phosphates (see 10°C reactions in Figure 6D versus Figures 2F and 3B).

Wild type ResT, V231A and E325A cleave the *rTel* with the +2 nick and form hp1 normally. hp2 forms very slowly due to the fact that after DNA cleavage the dinucleotide 3′ to the top strand scissile phosphate diffuses away producing a strand that is only four nucleotides long and lacks self-complementarity for subsequent foldback and strand joining. Unexpectedly, the Q161A and especially the D328A mutants form hp2 despite the short strand length and lack of dyad symmetry of the refolding strand (Figure 6B). In principle, the ability of these two mutants to form hp2 could be the result of an enhanced ability to rejoin folded strands that lack the ability to form base pairs at the site of strand rejoining or, alternatively, they could have the ability to cleave the DNA at a different site that yields a self-complementary strand that can fold and rejoin normally. We ruled out altered cleavage site selection by measuring the lengths of hp2 on sequencing gels (Figure 6B; denaturing gel panel). The length of hp2 formed with these mutants is the same as that formed at low levels with wild type ResT indicating that the Q161A and D328A mutants behave with the +2 nick *rTel* like they have reduced fidelity at the DNA rejoining step.

### ResT mutants with reduced hp formation fidelity

**Figure 7.**
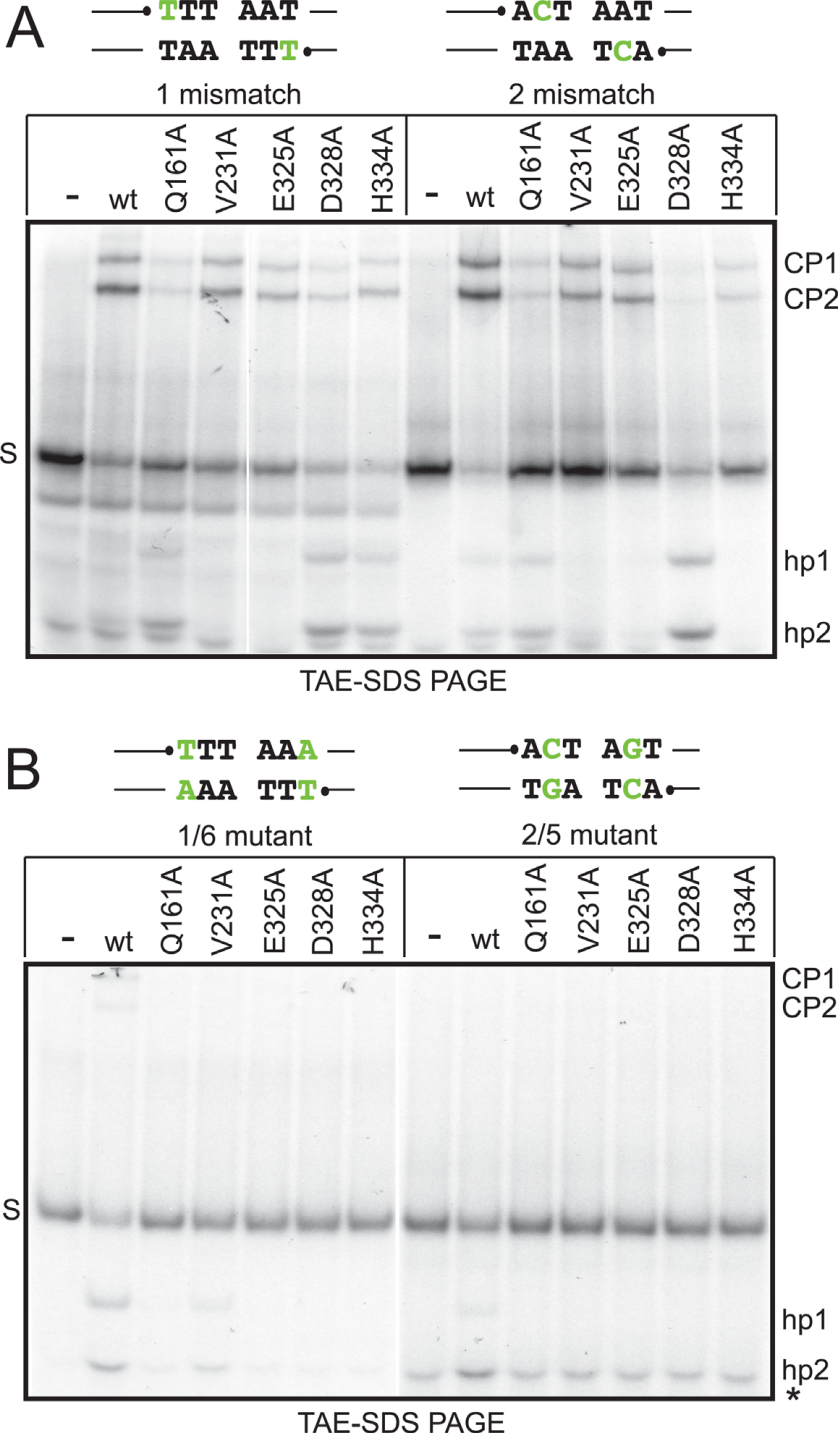
Assessing the fidelity of hairpin telomere formation with substrates with mismatches that inhibit hp formation. (**A**) 8% PAGE 1× TAE/0.1% SDS gel analysis of telomere resolution reactions incubated at 30°C for 10 min using Type 2 *rTels* with 1 mismatch and 2 mismatch modifications. The sequence of the modified *rTels* is shown above the gel with the nucleotide changed to create the mismatches represented in green/shaded script. The ResT variants assayed are indicated in the loading key above the gel. Gel labels are as described for Figure 6 except CP1 represents the cleavage product derived from the left side of the substrate. See Figure 2A and B for substrate and reaction details. (**B**) 8% PAGE 1× TAE/0.1% SDS gel analysis of telomere resolution reactions incubated at 30°C for 10 min using Type 2 *rTels* with 1/6 AT to TA and 2/5 TA to CG mutations that return full basepairing to the substrates but maintain the sequence change present in the mismatched substrates used in (**A**). The sequence of the mutant *rTels* is shown above the gel with the nucleotides changed to create the mutations represented in green/shaded script. Gel labels are as described previously except *, indicates unannealed oligo present in unreacted substrate.

In order to test if the observed reduction in the fidelity of hp formation was particular to reactions with the +2 nick *rTel* the mutants were re-assayed with substrates with intentionally engineered mismatches, at or near the site of strand rejoining, that inhibit hp telomere formation (Figure 7). Wild-type ResT and some of the mutants accumulate cleavage products due to the inhibition of hp telomere formation (CP1 and CP2 in Figure 7B). Mutants with reduced hp formation fidelity accumulated fewer cleavage products due to an enhanced ability to subsequently form hp telomeres (hp1 and hp2 in Figure 7B). In particular, the Q161A and D328A mutants demonstrated an enhanced ability to form hp telomeres, with both the 1 mismatch and 2 mismatch substrates, relative to other ResT variants. ResT (H334A) demonstrated reduced hp formation fidelity specific to the 1 mismatch *rTel* (Figure 7B). Since all the mutants in this study were initially selected as defective for telomere resolution on substrates with normal base pairing, but were rescuable by abasic modifications that mimic DNA unwinding between the scissile phosphates, we tested the activity of the mutants on control mutant *rTel*s that introduced compensatory mutations to restore base pairing (Figure 7C). This analysis verified that the activity of the mutants seen with the mismatch substrates was due to the introduction of the mismatches. An exception was the small degree of activity of the V231A mutant seen with the 1/6 control mutant. In no case was cleavage product accumulation seen without the mismatches (Figure 7C). The hp telomere products produced by the altered fidelity mutants were visualized on denaturing sequencing gels to ensure that the size of the hairpin telomeres were the same as produced from the unmodified *rTel* by wild type ResT (data not shown). This ruled out altered cleavage site selection.

## DISCUSSION

### Spring loading a pre-cleavage intermediate for hairpin telomere formation

**Figure 8.**
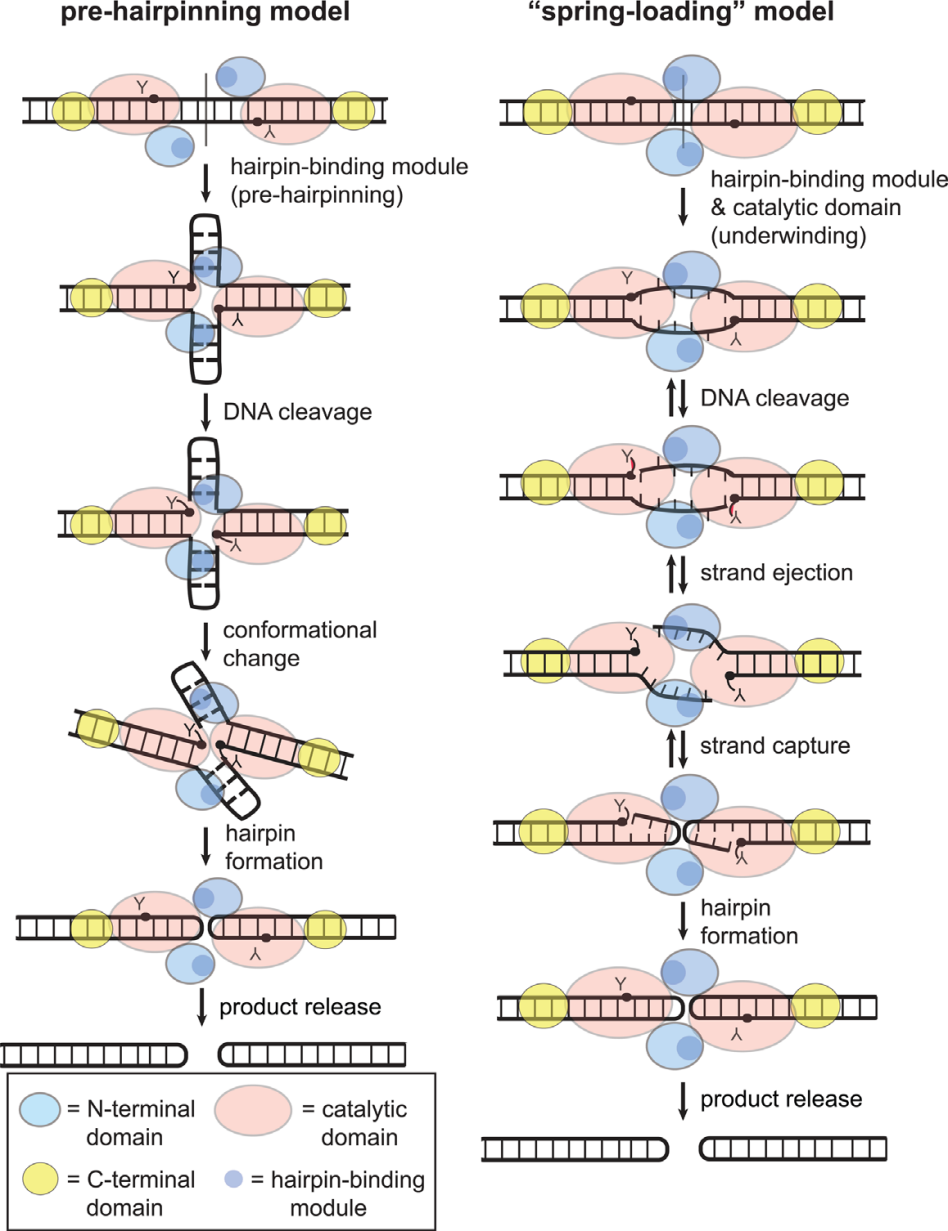
Comparison of pre-hairpinning and spring-loading models of ResT-mediated telomere resolution. The **pre-hairpinning model**, initially reported, proposed a distortion of the substrate DNA between the scissile phosphates that preforms hairpins prior to DNA cleavage (23). Similarity of the last ∼20 amino acids of the N-terminal domain to IS4 family transposases that excise transposons via a DNA hairpin intermediate, prompted this area of the ResT to be called the hairpin-binding module. In this model the hairpin-binding module creates the pre-hairpinned structure and the catalytic domain is restricted to DNA cleavage and rejoining activities. Most mutants in the hairpin-binding module display a cold-sensitivity that is rescued by heteroduplexing the central two base pairs of the *rTel*. Proposition of the pre-hairpinning model was motivated by the finding that hairpin-binding module mutants were cleavage defective rather than just defective for hp telomere formation (23). This model does not predict reversibility of the reaction after DNA cleavage. The **spring-loading model**, based on this report, similarly hypothesizes a distortion of the substrate DNA between the scissile phosphates that promotes DNA cleavage. Here the proposed distortion is an underwinding of the DNA. Breaking the 1/6 and 2/5 base pairs promotes subsequent strand ejection once the DNA is cleaved. The hypothesized underwound or ‘spring loaded’ pre-cleavage intermediate is formed by cooperation of the hairpin-binding module and the catalytic domain. The underwound conformation promotes strand ejection and the forward direction of the reaction. Our discovery of hp formation fidelity mutants suggests the presence of a reversible hp capture step in the reaction. The Q161, D328 and H334 residues are used to read out hp capture.

An early indication that ResT distorts the substrate DNA between the scissile phosphates, prior to DNA cleavage, derived from studies of mutants in the hairpin-binding module. Mutation of hairpin-binding module residues produced mutants that had difficulty initiating DNA cleavage, especially at reduced reaction temperature (23). This phenotype was rescued by heteroduplexing the central 2 base pairs of the *rTel*. This was interpreted as telomere resolution featuring a, pre-cleavage, refolding of the DNA between the scissile phosphates into hairpin structures (pre-hairpinning model of Figure 8, (23)). The pre-hairpinning model does not predict reversibility of the reaction after DNA cleavage. A subsequent study, of product release during telomere resolution, suggested that there must be a reversible component to the reaction after DNA cleavage. This conclusion was drawn from substrates engineered to form the 2 hp telomeres at different rates; hp telomere formation and release slowed to that of the slow forming hp telomere (15). The model of the pre-cleavage distortion of the *rTel* was refined to an undefined unwinding of the substrate DNA between the cleavage sites, induced by the hairpin-binding module. This model for ResT is presented in Figure 1. The current study sheds detailed light on the positions at which the proposed substrate unwinding occurs, and demonstrates that the hairpin-binding module and the catalytic domain cooperate in forming the underwound pre-cleavage intermediate.

Our current working model of telomere resolution by ResT features, like TelK, a ‘spring loaded’ pre-cleavage intermediate and, like TelA, ResT-mediated strand refolding in the context of a dimer of ResT (see Figures 1 and 8). Our finding that breaking base pairs between the scissile phosphates, both through missing base and mismatch modifications, stimulates DNA cleavage and alleviates the cold-sensitivity of telomere resolution, points to unwinding the substrate DNA in this region as a key pre-cleavage cold-sensitive step of the reaction. This is consistent with previous results that show that ResT can run the reverse reaction, fusing hp telomeres together, and that this reaction can run at low temperature (8°C). By contrast, the forward reaction quickly becomes inoperative <25°C (8,19). The pre-melting of the DNA, expected to be present in the very tip of the hp telomeres, was inferred to be responsible for the lack of cold-sensitivity of the reverse reaction.

Between this study and that of Bankhead and Chaconas (23), there have now been 13 residues in ResT, distributed throughout the hairpin-binding module and now the catalytic domain, implicated in stabilizing an underwound pre-cleavage intermediate. This study highlights the particular importance of breaking and stabilizing the unpaired bases from the 1/6 and 2/5 base pairs (Figures 4 and 7). The proposed pre-cleavage intermediate is modeled as ‘spring loaded’, or as storing the energy needed, after DNA cleavage, to eject the strands away from the 3′-phosphotyrosine cleavage intermediate, preventing regeneration of substrate and promoting strand refolding for hp telomere formation (Figure 8). In the original model of such a pre-cleavage intermediate, the DNA distortion was modeled as a pre-formation of the hairpins before DNA cleavage commenced. However, the properties of the mutants characterized in this study lead us to favor a substrate unwinding, strand ejection and reversible hp capture model (Figure 8). The results of missing base rescue, of the mutants in this study, implicate stabilization of an extrahelical conformation of the bases in the 2/5 base pairs as key in the unwinding step (Figure 4). Similar to the phenotype of hairpin-binding module mutants, the Q161A, V231A and H334A mutants were found to be cleavage defective on unmodified *rTel*. In contrast, the E325A and D328A mutants appear to undergo abortive cycles of cleavage and rejoining. Absence of the acidic sidechains at these positions seems to attenuate the energy required to eject the cleaved strands away from the ResT-DNA phosphotyrosyl linkages. This ‘ejection energy’ is needed to prevent the rapid strand rejoining that would regenerate substrate (Figure 5). Breaking the base pairing near the scissile phosphates, particularly by removal of the base at position 2, can partially reduce the need for this ejection energy (Figure 4).

Evidence for a reversible hp capture step is afforded by the unexpected hp formation fidelity phenotype of some of the mutants (Q161A, D328A and H334A). The identification of mutants with an enhanced ability to form hairpin telomeres from substrates with inappropriate strand lengths and/or the lack of base pairing potential at the site of strand joining implies that ResT, directly or indirectly, reads out base pair formation during hp capture. It would seem a mild barrier to strand rejoining is normally established. Refolding DNA strands that cannot be stabilized by base pair formation in the developing hp, regenerate substrate. The acidic D328 residue seems important for this reversibility; the cleavage competent D328A mutant allows proficient hp formation from strands with inappropriate lengths and from strands lacking self-complementarity (Figures 6 and 7). A mechanism of telomere resolution with a reversible hp capture step would help guard against frequent deleterious fragmentation of the genome by attempted resolution at sites that are not *bona fide* replicated telomeres (Figure 8).

### Generality of the spring loading model?

Our proposal of a spring loaded pre-cleavage intermediate has similarities to that proposed for the TelK system, in that the intermediate is proposed to control reaction directionality (Figure 1). However, how the pre-cleavage intermediate is formed and how it determines reaction directionality in the two models is entirely different. For TelK, an out-of-plane DNA bending, established without contacts between TelK and the DNA between the cleavage sites, is proposed to drive dimer dissolution. This allows subsequent strand refolding and joining to form the hp telomeres (Figure 1). The dissolution of the dimer preceding hp formation is proposed to act as a ‘kinetic trap’ propelling the reaction toward product formation (17). The inability of TelK to promote hp telomere fusion, in contrast to ResT, provides further evidence of this essential difference (17,19).

The elegant structural studies of TelA captured snap shots of TelA's role in stabilizing the DNA in the strand refolding and hp telomere product complexes. These data are not incompatible with TelA also possessing a spring loaded pre-cleavage intermediate. Functional studies will be needed to test this. Similarly, a spring loaded pre-cleavage intermediate for telomere resolution by ResT is not incompatible with the possible existence of ResT-stabilized refolding intermediates. Structural studies similar to those performed for TelA will be needed to test this possibility.

## SUPPLEMENTARY DATA

Supplementary Data are available at NAR Online.

SUPPLEMENTARY DATA
