# Supplementary material for: Spring loading a pre-cleavage intermediate for hairpin telomere formation
Source: Nucleic Acids Res. 2015 May 24;43(12):6062–74. doi: 10.1093/nar/gkv497 (PMC4499125; doi:10.1093/nar/gkv497)
Supplement: SUPPLEMENTARY DATA [file supp_43_12_6062__index.html]

Spring loading a pre-cleavage intermediate for hairpin telomere formation — Spring loading a pre-cleavage intermediate for hairpin telomere formation — SUPPLEMENTARY DATA 

# Spring loading a pre-cleavage intermediate for hairpin telomere formation

## SUPPLEMENTARY DATA

- SUPPLEMENTARY DATA
