# Supplementary material for: Spring loading a pre-cleavage intermediate for hairpin telomere formation
Source: Nucleic Acids Res. 2015 May 24;43(12):6062–74. doi: 10.1093/nar/gkv497 (PMC4499125; doi:10.1093/nar/gkv497)
Supplement: SUPPLEMENTARY DATA [file supp_gkv497_nar-03702-f-2014-File010.pdf]

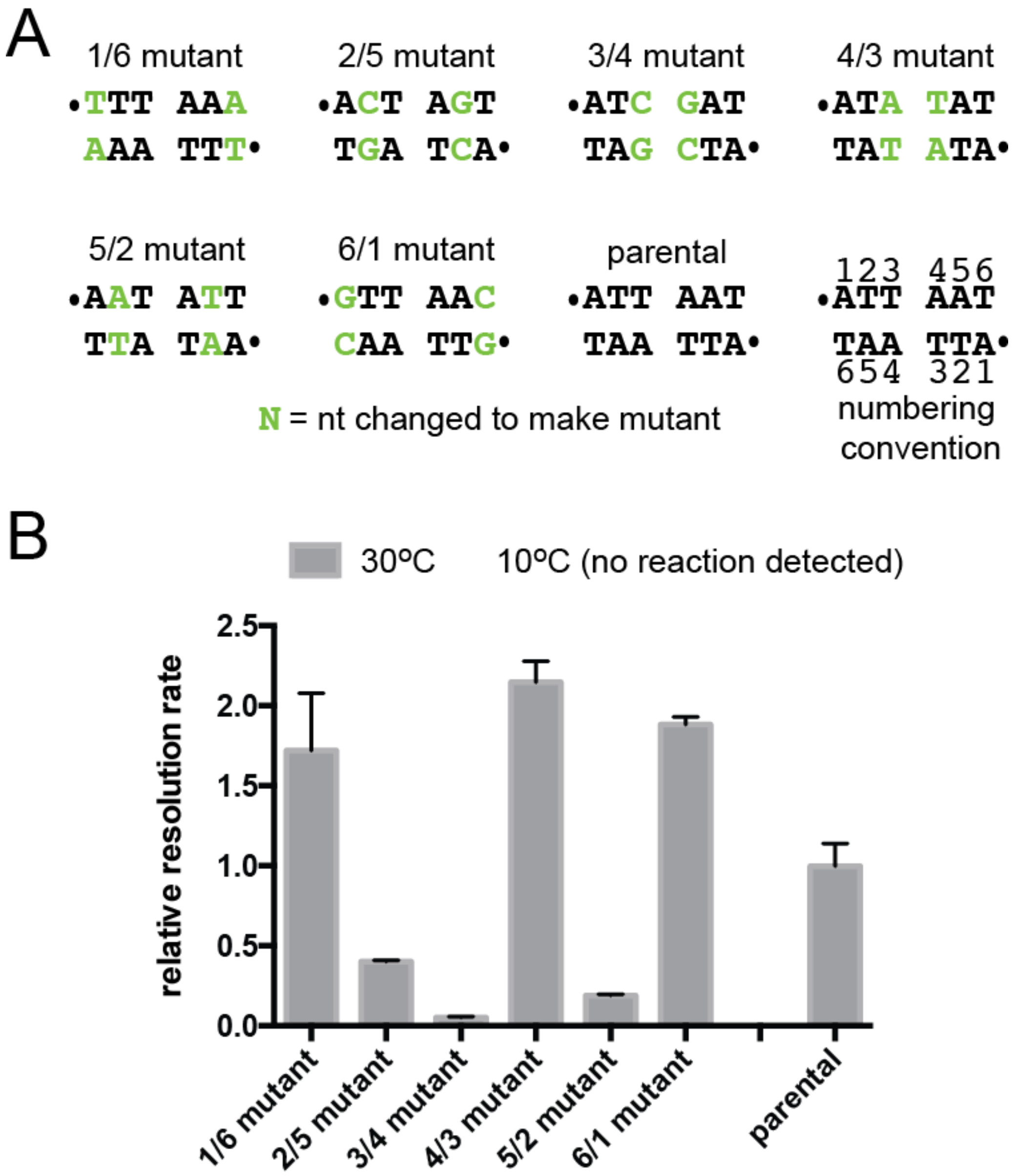

Figure S1. Control Type 2 mutant *rTels* do not alleviate the cold-sensitivity of telomere resolution. A) Schematic representation of the sequence between the scissile phosphates of the parental Type 2 *rTel* and the range of tested substrate *rTe*ls with mutations in symmetric locations on the top and bottom strands. The nucleotide substitutions that generate the mutations are represented in the sequence by the green shaded letters and the scissile phosphates by dots. These mutant *rTel*s harbour compensatory mutations that return full base pairing to the mismatch *rTel*s used in Figures 3 & 7.

B) Comparison of the relative rate of telomere resolution, assayed at 30°C vs. 10°C incubation temperatures, using the mutant *rTel*s outlined in A) normalized to the rate of telomere resolution measured with the parental *rTel* substrate. There was no detectable reaction with any of the tested *rTel*s in 10°C incubations. Shown is the mean and standard deviation of at least 3 independent replicates.

A

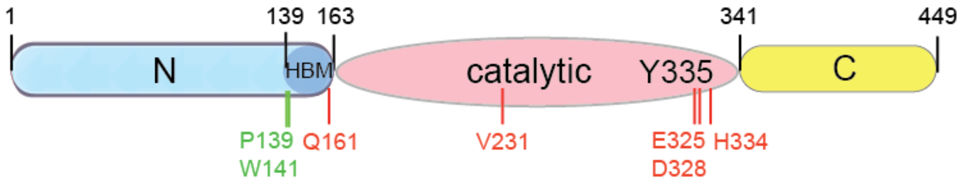

B

mutant	purified	tel. res.	tel. res. rescue	tel. binding	domain
H46A	no	NA	NA	NA	N
H147A	yes	cold sensitive	yes	normal	N (HBM)
Y149A	yes	cold sensitive	yes	normal	N (HBM)
E160A	yes	normal	NA	normal	N (HBM)
Q161A	yes	inactive	yes	normal	N (HBM)
Q181A	yes	normal	NA	normal	catalytic
H216A	yes	normal	NA	normal	catalytic
N229A	yes	normal	NA	normal	catalytic
I230A	no	NA	NA	NA	catalytic
V231A	yes	inactive	yes	normal	catalytic
H324A	yes	inactive	no	normal	catalytic
E325A	yes	inactive	yes	normal	catalytic
D328A	yes	inactive	yes	normal	catalytic
H334A	yes	inactive	yes	normal	catalytic

C

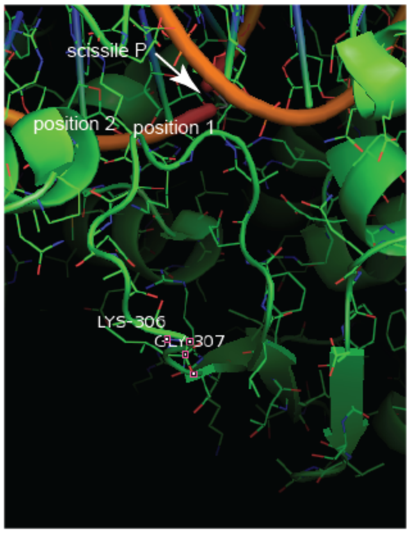

ResT (V231) analogue in TelK-cleaved *rTel* DNA structure is TelK (G307)

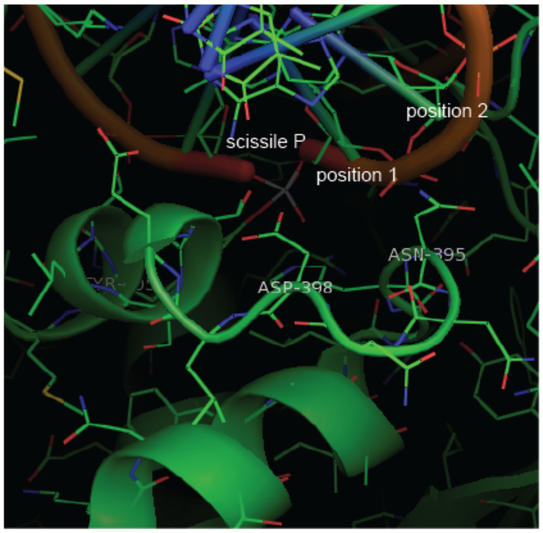

ResT (E325) and ResT (D328) analogues in the TelA-hp telomere structure are TelA (N395) and (D328)

Figure S2. Summary of ResT mutants examined for this study.
A) This schematic presents the position of the alanine mutants, reported in this study, overlaid on a representation of the domain organization of ResT. Previous mutants implicated in distorting the DNA between the scissile phosphates, fall in the hairpin-binding module (HBM) at the end of the N-terminal domain (residues 139-163). The position of the residues with the strongest previously reported mutant phenotype (P139 and W141) are indicated in green script under the schematic, while the mutants characterized in this study are indicated in red script.

B) Table of mutants generated for this study. We performed a mutagenic survey of all the histidine and glutamine residues in ResT that are conserved across both relapsing fever and Lyme disease causing *Borrelia* species. Y149A was generated to test if it was a functional analogue of TelA (Y201) which is crucial for hp telomere formation in that system. This mutant shared the cold-sensitivity shared by many other mutants in the ResT hairpin-binding module, including H147A first reported here (23). Finally, based on the structures of TelA and TelK, we noted that coils between β–β and α-α secondary elements track on opposite sides of the double helix near the scissile phosphates. We hypothesized that these loops would be in position to interact with the DNA in the underwound pre-cleavage intermediate. Alanine mutants of polar or hydrophobic residues in loop 1 (N229, I230 and V231) and acidic residues in loop 2 (E325 and D328) were generated. In the table is indicated whether the generated mutant could be purified, was active in telomere resolution assays, telomere binding assays, whether the mutant could be rescued by modifications that destabilize the DNA between the scissile phosphates and the ResT domain the residue falls in. Details of the screening procedure and criteria used for inclusion of mutants in this study are available in the Supplementary Material and Methods. NA = not applicable.
C) Positioning the ResT residues mutated in this study in the TelK and TelA structures. Annotated are the positions of the scissile phosphates, position 1 and 2 in the substrate DNA and the residue position and numbering derived from the structure noted below each graphic. The structure chosen for the residue positioning was determined by which protein had similar sequence element spacing in the appropriate region of the alignments. V231 exists on a coil between β elements in the catalytic domain that is the same length in ResT and TelK, TelA has a 6 amino acid insertion in the analogous position. ResT residues E325, D328 and H334 lie on a coil between α elements in a region of the catalytic domain where ResT and TelA have a 2 amino acid insertion relative to the spacing of conserved active site residues found in all 3 telomere resolvases (10). The structures presented were generated with The PyMOL Molecular Graphics System, Version 1.7 Schrödinger, LLC. (http://www.pymol.org/) using PDB ID 2v6e for TelK and 4e0g for TelA (16,17).

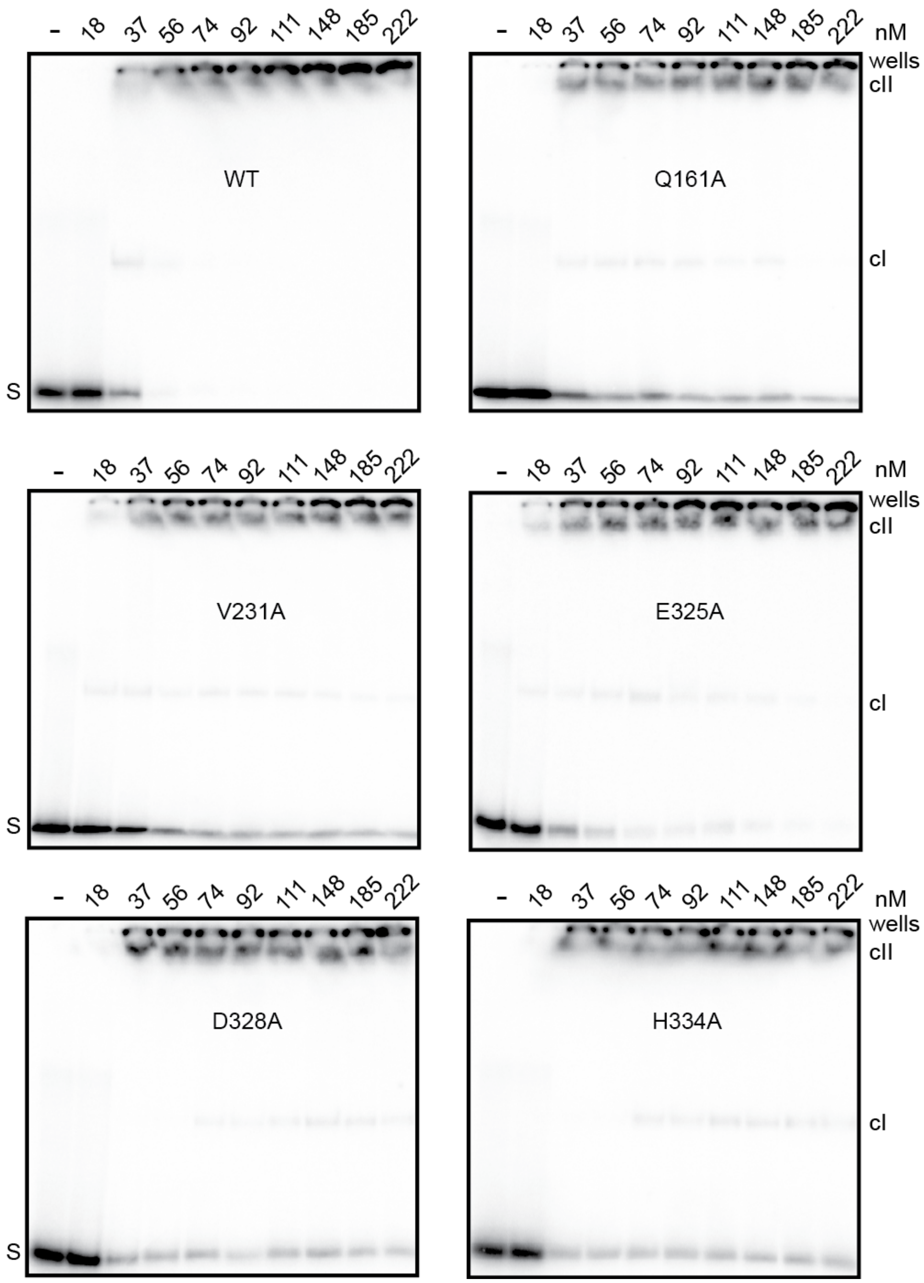

Figure S3. Electrophoretic mobility shift assays (EMSA) of the ResT variants used in this study. 5'-^{32}P endlabeled half-site telomere with a 5'-single-stranded overhang assembled from oligonucleotides OGCB124/120 was used in EMSAs (Supplemental Material and Methods). The substrate was present at 5.25 nM, and was incubated with the indicated concentration of ResT at 0°C for 20 min in a buffer containing 25 mM Tris-HCl (8.5), 1 mM EDTA (8.0), 100 μg/ml BSA, 78 ng/ml heparin sulphate and 100 mM NaCl. The binding reactions were loaded onto 6% PAGE 0.5 X TBE gels run at 15 V/cm and 4°C until the bromophenol blue tracking dye was 3 cm from the bottom of the gel. To load the binding reactions to the gels load dye was added to a final concentration of 1X (10 X contains 200 mM EDTA, 32% glycerol, 0.024% bromophenol blue). Gels were dried for autoradiography as reported (Material and Methods). The EMSAs are characterized by shifts to two complexes that enter the gel (cI and cII) presumably reflecting different oligomeric forms of ResT bound to the substrate (S). At higher concentrations ResT is prone to producing a well shift (labeled on the gels as wells).

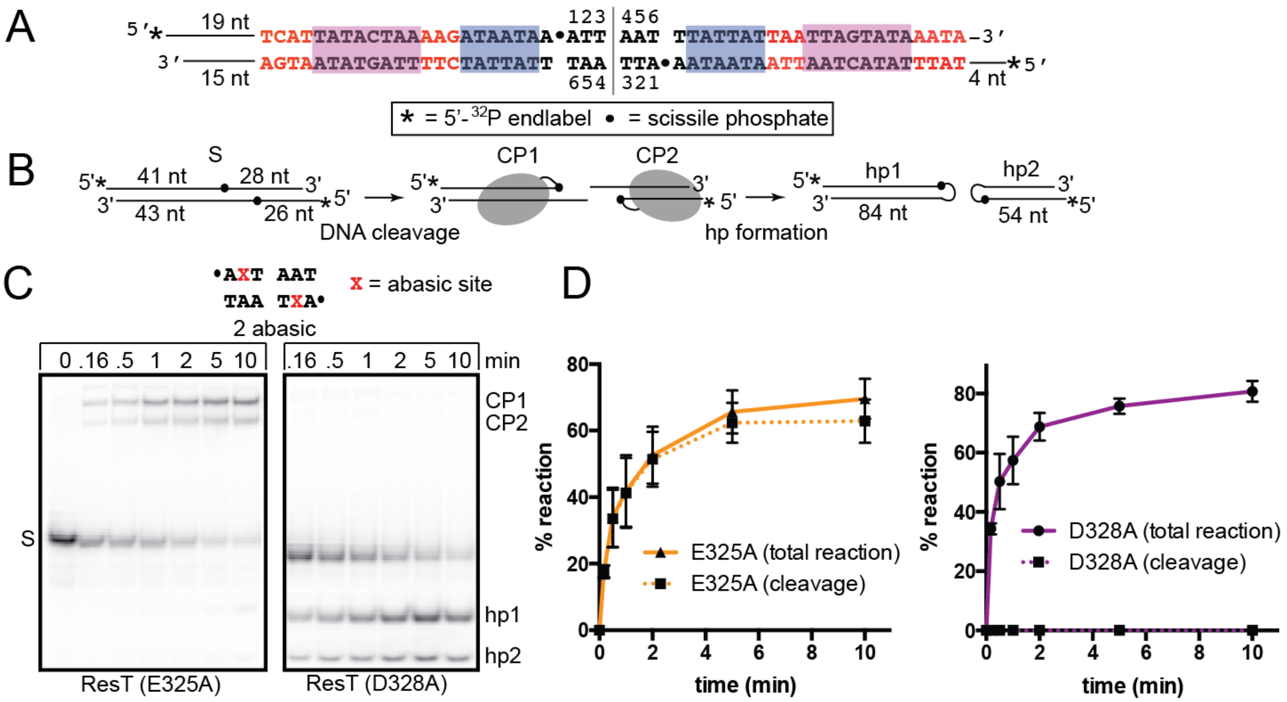

Figure S4. Initial rate determination for telomere resolution reactions with mutant ResTs and *rTels* with missing bases.

A) The model Type 2 *rTel* used in this study.

B) Schematic representation of a telomere resolution reaction with 5'-^{32}P endlabeled substrate.

C) Representative 8% PAGE 1XTAE/0.1% SDS gels of timecourse reactions with ResT (E325A) and ResT (D328A) with the 2 abasic version of the substrate detailed in A) and represented above the gels. S, represents the mobility in the gel of the substrate; CP1 & 2, mark the gel migration position of the cleavage products; hp1 & hp2, mark the gel position of the hairpin telomere products detailed in B).

D) % reaction (DNA cleavage and total reaction) vs. reaction time is plotted for a telomere resolution reaction with ResT (E325A) and the 2 abasic *rTel* incubated at 30°C (left graph). % reaction (DNA cleavage and total reaction) vs. reaction time is plotted for a telomere resolution reaction with ResT (D328A) and the 2 abasic *rTel* incubated at 30°C (right graph). Shown are the mean and standard deviation of at least 3 independent replicates. ResT (E325A) produces primarily cleavage products (dotted line) with very slow conversion to hairpin telomeres (hp formation + cleavage; solid line). Conversely, ResT (D328A) converts straight to hairpin telomere products accumulating little cleavage product. Here, the total reaction is derived from hp formation. Initial rates shown in Figure 4 were determined from individual % reaction vs. time plots by determination of the slope of the initial linear portion of the curves; means and standard deviations for the initial rates were determined from at least 3 independent timecourses.

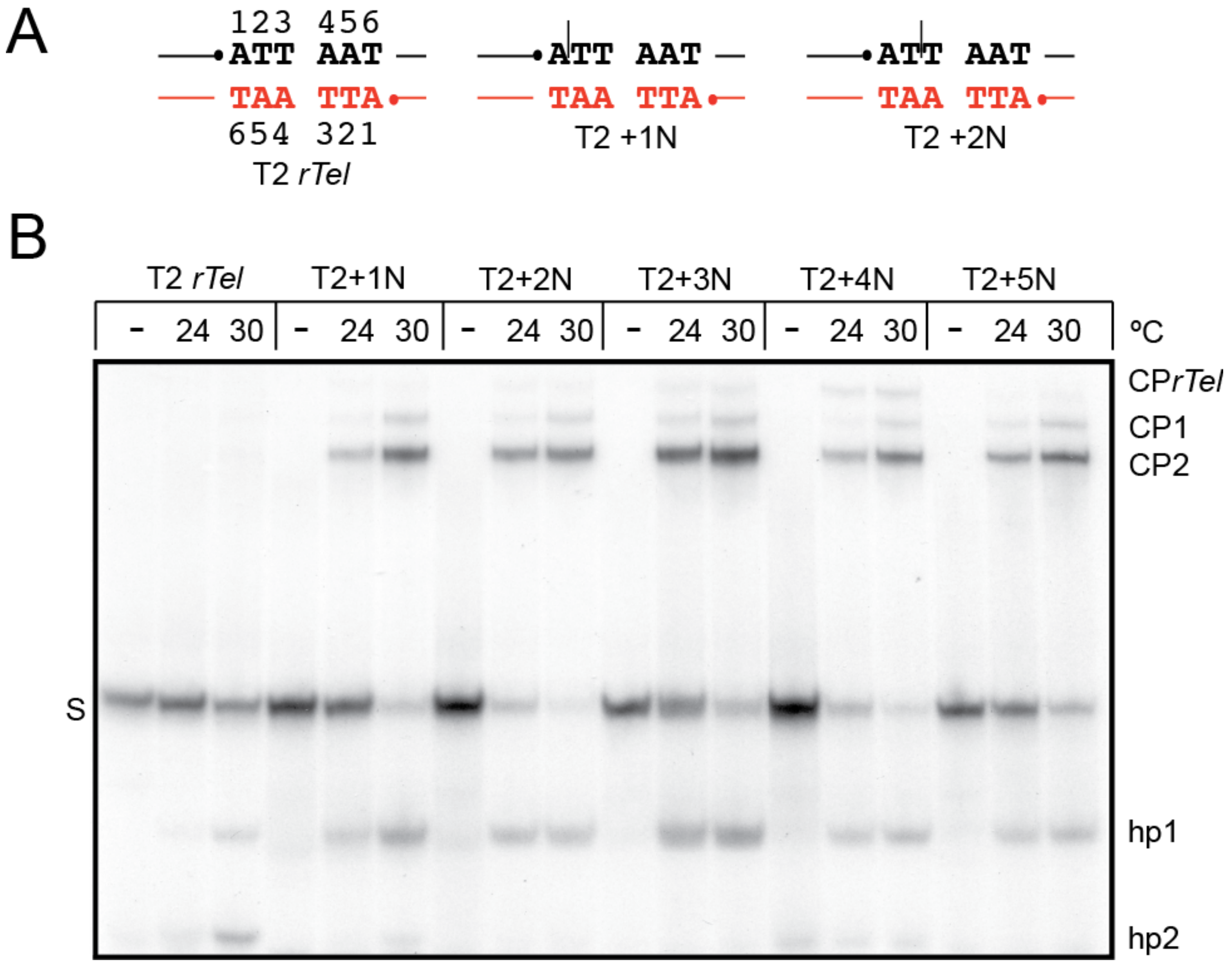

Figure S5. Reactivity of Type 2 *rTels* with nicks at various positions between the scissile phosphates.

A) Schematic representation of the sequence of the Type 2 *rTel* between the scissile phosphates (represented by the dots). The numbering convention used is the same as that used for the TelA-DNA and TelK-DNA structures. The vertical line interrupting the top strand sequence represents a missing phosphate (a nick). Examples of +1 and +2 nicked *rTel*s are shown (T2+1N and T2+2N).

B) A representative 8% PAGE 1X TAE/0.1% SDS gel analysis of telomere resolution reactions performed with 75 nM wild type ResT and 5.25 nM of the indicated Type 2 and nicked Type 2 *rTels*. The reactions were incubated at 24°C for 30 min or at 30°C for 15 min. S, represents the migration position in the gel of the substrate *rTels*; CP1 and CP2, represent the migration position in the gel of ResT-DNA cleavage intermediates that would give rise to hp1 and hp2, respectively on an unmodified *rTel*; CP*rTel*, represents a gapped *rTel* cleavage product resulting of cleavage of the top strand only; hp1 and hp2, represent the migration position in the gel of the hairpin telomere products.

Supplementary Material and Methods

ResT mutant creation and screening

ResT mutants, listed in Figure S2, were generated by site-specific mutagenesis. The mutants were expressed and purified as noted in (22). Preliminary telomere resolution proficiency screening was conducted with a 5'-^{32}P endlabeled model Type 1 *rTel* in 10 min reactions incubated at 30°C under standard buffer conditions (25 mM Tris-Cl, pH 8.5; 1 mM EDTA, pH 8.0; 100 μg/ml BSA; 100 mM NaCl) using 37, 74 and 148 nM concentrations of ResT. Mutants with normal reactivity at 30°C were additionally tested as above in reactions incubated at 20°C to test for a cold-sensitivity phenotype typical of the majority of hairpin-binding module mutants (23). Mutants with a mild cold-sensitive phenotype fell within the hairpin-binding module and were not investigated further (H147A and Y149A). Mutants that were inactive in the initial screen at 30°C were then tested in 10 min reactions incubated at 30°C using a Type 1+2N nicked telomere using the above noted ResT concentration range. Rescue of inactivity by the +2N nick substrate marked these mutants as candidates for inclusion in the study. Finally, because the decision was made to use a Type 2 *rTel* and modified versions of this parent the mutants were rescreened as above to ensure that none of the mutants were specifically defective on the Type 1 *rTel* used for the activity screen. The mutants in Figure S2 that were generated but not purified or assayed were insoluble in expression trials conducted at 24°C and 16°C and were not investigated further (H46A and I230A).

***rTel* substrate assembly and 5'-^{32}P endlabeling**

The *rTel* substrates, except the nicked *rTel*s, were assembled by 5'- ^{32}P endlabeling the oligos with T4 polynucleotide kinase (PNK) and γ^{32}P-ATP followed by annealing (37°C, 1 hour, using, 66 nM γ^{32}P-ATP and 4 units of T4 PNK). The nicked *rTel*s were annealed first and the annealed substrates were 5'- ^{32}P endlabeled at 30°C, for 1 hour, with 4 units of T4 PNK and 66 nM γ^{32}P-ATP. Under these conditions, T4 PNK does not detectably label the internal nick. This leaves a 5'-OH at the position of the nick. This is potentially competent for hp formation. The OPS *rTel* is dyad symmetric and must, therefore, be assembled as halfsites that are then ligated and gel purified as reported in reference 23.

Table S1. Oligonucleotides used to make the substrates used in this study.

Oligo name	Oligo sequence	Substrate
OGCB127	5'-GATCtcctctaaccattgcTCATTATACTAAAAGATAATAAATTAAT TTATTATTAATTAGTATAAATA-3'	Type 2 *rTel*
OGCB128	5'-GATCTATTTATACTAATTAATAATAAATTAATTTATTATCTTTTAGT ATAATGAgcaatggttagagga-3'	Type 2 *rTel*
OGCB242	5'-GATCtcctctaaccattgcTCATTATACTAAAAGATAATAAXTT AATTTATTATTAATTAGTATAAATA-3'	1 abasic
OGCB248	5'-GATCTATTTATACTAATTAATAATAAXTT AATTTATTATCTTTTAGTATAATGAgcaatggttagagga-3'	1 abasic
OGCB356	5'-GATCtcctctaaccattgcTCATTATACTAAAAGATAATAATXA TAATTATTATTAATTAGTATAAATA-3'	2 abasic
OGCB363	5'-GATCTATTTATACTAATTAATAATAATXA TAATTATTATCTTTTAGTATAATGAgcaatggttagagga-3'	2 abasic

OGCB357	5′-GATCtcctctaaccattgcTCATTATACTAAAAGATAATAATTX TAATTATTATTAATTAGTATAAATA-3'	3 abasic
OGCB362	5′-GATCTATTTATACTAATTAATAATAATTX TAATTATTATCTTTTAGTATAATGAgcaatggttagagga-3′	3 abasic
OGCB358	5′-GATCtcctctaaccattgcTCATTATACTAAAAGATAATAATTA XAATTATTATTAATTAGTATAAATA-3'	4 abasic
OGCB361	5′-GATCTATTTATACTAATTAATAATAATTA XAATTATTATCTTTTAGTATAATGAgcaatggttagagga-3′	4 abasic
OGCB359	5′-GATCtcctctaaccattgcTCATTATACTAAAAGATAATAATTA TXATTATTATTAATTAGTATAAATA-3'	5 abasic
OGCB360	5′-GATCTATTTATACTAATTAATAATAATTA TXATTATTATCTTTTAGTATAATGAgcaatggttagagga-3′	5 abasic
OGCB244	5′-GATCtcctctaaccattgcTCATTATACTAAAAGATAATAAATT AAXTTATTATTAATTAGTATAAATA-3'	6 abasic
OGCB246	5′-GATCTATTTATACTAATTAATAATAAATT AAXTTATTATCTTTTAGTATAATGAgcaatggttagagga-3′	6 abasic
OGCB241	5′-GATCtcctctaaccattgcTCATTATACTAAAAGATAATAATTT AATTTATTATTAATTAGTATAAATA-3'	1 mismatch
OGCB247	5′-GATCTATTTATACTAATTAATAATAATTT AATTTATTATCTTTTAGTATAATGAgcaatggttagagga-3′	1 mismatch
OGCB197C	5′-GATCtcctctaaccattgcTCATTATACTAAAAGATAATAAACT AATTTATTATTAATTAGTATAAATA-3'	2 mismatch
OGCB204C	5′-GATCTATTTATACTAATTAATAATAAACT AATTTATTATCTTTTAGTATAATGAgcaatggttagagga-3′	2 mismatch
OGCB198C	5′-GATCtcctctaaccattgcTCATTATACTAAAAGATAATAAATC AATTTATTATTAATTAGTATAAATA-3'	3 mismatch
OGCB203C	5′-GATCTATTTATACTAATTAATAATAAATC AATTTATTATCTTTTAGTATAATGAgcaatggttagagga-3′	3 mismatch
OGCB199T	5′-GATCtcctctaaccattgcTCATTATACTAAAAGATAATAAATT TATTTATTATTAATTAGTATAAATA-3'	4 mismatch
OGCB202T	5′-GATCTATTTATACTAATTAATAATAAATT TATTTATTATCTTTTAGTATAATGAgcaatggttagagga-3′	4 mismatch
OGCB200T	5′-GATCtcctctaaccattgcTCATTATACTAAAAGATAATAAATT ATTTTATTATTAATTAGTATAAATA-3'	5 mismatch
OGCB201T	5′-GATCTATTTATACTAATTAATAATAAATT ATTTTATTATCTTTTAGTATAATGAgcaatggttagagga-3′	5 mismatch
OGCB243	5′-GATCtcctctaaccattgcTCATTATACTAAAAGATAATAAATT AACTTATTATTAATTAGTATAAATA-3'	6 mismatch
OGCB245	5′-GATCTATTTATACTAATTAATAATAAATT AACTTATTATCTTTTAGTATAATGAgcaatggttagagga-3′	6 mismatch
OGCB617	5′-GATCtcctctaaccattgcTCATTATACTAAAAGATAATAATTT AAATTATTATTAATTAGTATAAATA-3'	1/6 mutant
OGCB618	5′-GATCTATTTATACTAATTAATAATAATTT AAATTATTATCTTTTAGTATAATGAgcaatggttagagga-3′	1/6 mutant
OGCB267	5′-GATCtcctctaaccattgcTCATTATACTAAAAGATAATAAACT AGTTTATTATTAATTAGTATAAATA-3'	2/5 mutant
OGCB268	5′-GATCTATTTATACTAATTAATAATAAACT AGTTTATTATCTTTTAGTATAATGAgcaatggttagagga-3′	2/5 mutant
OGCB269	5′-GATCtcctctaaccattgcTCATTATACTAAAAGATAATAAATC GATTTATTATTAATTAGTATAAATA-3'	3/4 mutant
OGCB270	5′-GATCTATTTATACTAATTAATAATAAATC GATTTATTATCTTTTAGTATAATGAgcaatggttagagga-3′	3/4 mutant
OGCB271	5′-GATCtcctctaaccattgcTCATTATACTAAAAGATAATAAATA TATTTATTATTAATTAGTATAAATA-3'	4/3 mutant
OGCB272	5′-GATCTATTTATACTAATTAATAATAAATA TATTTATTATCTTTTAGTATAATGAgcaatggttagagga-3′	4/3 mutant

OGCB273	5′-GATCtcctctaaccattgcTCATTATACTAAAAGATAATAAAAT ATTTTATTATTAATTAGTATAAATA-3'	5/2 mutant
OGCB274	5′-GATCTATTTATACTAATTAATAATAAAAT ATTTTATTATCTTTTAGTATAATGAgcaatggttagagga-3′	5/2 mutant
OGCB619	5′-GATCtcctctaaccattgcTCATTATACTAAAAGATAATAAGTT AACTTATTATTAATTAGTATAAATA-3'	6/1 mutant
OGCB620	5′-GATCTATTTATACTAATTAATAATAAGTT AACTTATTATCTTTTAGTATAATGAgcaatggttagagga-3′	6/1 mutant
OGCB129	5′-GATCtcctctaaccattgcTCATTATACTAAAAGATAATAAA-3′	Type 2+1N
OGCB130	5′-TT AATTTATTATTAATTAGTATAAATA-3′	Type 2+1N
OGCB128	5′-GATCTATTTATACTAATTAATAATAAATT AATTTATTATCTTTTAGTATAATGAgcaatggttagagga-3′	Type 2+1N
OGCB131	5′-GATCtcctctaaccattgcTCATTATACTAAAAGATAATAAAT-3′	Type 2+2N
OGCB132	5′-T AATTTATTATTAATTAGTATAAATA-3′	Type 2+2N
OGCB128	above	Type 2+2N
OGCB133	5′-GATCtcctctaaccattgcTCATTATACTAAAAGATAATAAATT-3′	Type 2+3N
OGCB134	5′-AATTTATTATTAATTAGTATAAATA-3′	Type 2+3N
OGCB128	above	Type 2+3N
OGCB135	5′-GATCtcctctaaccattgcTCATTATACTAAAAGATAATAAATT A-3′	Type 2+4N
OGCB136	5′-ATTTATTATTAATTAGTATAAATA-3′	Type 2+4N
OGCB128	above	Type 2+4N
OGCB137	5′-GATCtcctctaaccattgcTCATTATACTAAAAGATAATAAATT AA-3′	Type 2+5N
OGCB138	5′-TTTATTATTAATTAGTATAAATA-3′	Type 2+5N
OGCB128	above	Type 2+5N
OGCB116OPS	5′-gatcCACTCTATACTAATAAAAAATTA*TATAT-3′	OPS *rTel*
OGCB117	5′-ATAATTTTTTATTAGTATAGAGTG-3′	OPS *rTel*
OGCB124	5′-GATCtcctctaaccattgcACTTTATACTAAATAAATATTATAT-3′	EMSA substrate
OGCB120	5′-GATCATTCTATACTAATTAAAAATTATAT ATATAATATTTATTTAGTATAAAGTgcaatggttagagga-3′	EMSA substrate

X indicates a dSpacer abasic modification * indicates the 5'-bridging phosphorothioate modification

Supplementary References

10. Kobryn, K. and Chaconas, G. (2014) Hairpin Telomere Resolvases. *Microbiol Spectrum*, **2**, MDNA-0023-2014.

16. Shi, K., Huang, W.M. and Aihara, H. (2013) An enzyme-catalyzed multistep DNA refolding mechanism in hairpin telomere formation. *PLoS Biol*, **11**, e1001472.

17. Aihara, H., Huang, W.M. and Ellenberger, T. (2007) An interlocked dimer of the protelomerase TelK distorts DNA structure for the formation of hairpin telomeres. *Mol Cell*, **27**, 901-913.

22. Mir, T., Huang, S.H. and Kobryn, K. (2013) The telomere resolvase of the Lyme disease spirochete, *Borrelia burgdorferi*, promotes DNA single-strand annealing and strand exchange. *Nucleic Acids Res*, **41**, 10438-10448.

23. Bankhead, T. and Chaconas, G. (2004) Mixing active site components: A recipe for the unique enzymatic activity of a telomere resolvase. *Proc. Natl. Acad. Sci. USA*, **101**, 13768-13773.
